# Identification of a Potent and Selective 5-HT_1A_ Receptor Agonist with *In Vitro* and *In Vivo* Antinociceptive Activity

**DOI:** 10.1021/acschemneuro.0c00289

**Published:** 2020-12-02

**Authors:** Pasquale Linciano, Claudia Sorbi, Antonella Comitato, Anna Lesniak, Magdalena Bujalska-Zadrożny, Agata Pawłowska, Anna Bielenica, Jolanta Orzelska-Górka, Ewa Kędzierska, Grażyna Biała, Simone Ronsisvalle, Silvia Limoncella, Livio Casarini, Elena Cichero, Paola Fossa, Grzegorz Satała, Andrzej J. Bojarski, Livio Brasili, Rita Bardoni, Silvia Franchini

**Affiliations:** † Department of Life Sciences, University of Modena and Reggio Emilia, Via Campi 103, 41125 Modena, Italy; ‡ Department of Biomedical, Metabolic and Neural Sciences, University of Modena and Reggio Emilia, Via Campi 287, 41125 Modena, Italy; § Department of Pharmacodynamics, Faculty of Pharmacy, Centre for Preclinical Research and Technology, Medical University of Warsaw, Banacha 1, 02-097 Warsaw, Poland; ∥ Department of Biochemistry, Medical University of Warsaw, Banacha 1, 02-097 Warsaw, Poland; ⊥ Department of Pharmacology and Pharmacodynamics, Faculty of Pharmacy with Division of Medical Analytics, Medical University of Lublin, Chodzki 4A, 20-093 Lublin, Poland; # Department of Drug Sciences, Medicinal Chemistry Section, University of Catania, Viale A. Doria 6, I-95125 Catania, Italy; ¶ Unit of Endocrinology, Department Biomedical, Metabolic, and Neural Sciences, University of Modena and Reggio Emilia, via G. Campi 287, 41125 Modena, Italy; □ Center for Genomic Research, University of Modena and Reggio Emilia, via G. Campi 287, 41125 Modena, Italy; ● Department of Pharmacy, Medicinal Chemistry Section, School of Medical and Pharmaceutical Sciences, University of Genova, Viale Benedetto XV 3, 16132 Genova, Italy; ▽ Department of Medicinal Chemistry, Maj Institute of Pharmacology, Polish Academy of Sciences, 12, Smętna Street, 31-343, Kraków, Poland

**Keywords:** Serotonin receptors, 5-HT_1A_R agonists, pain, behavioral profiling, mice

## Abstract

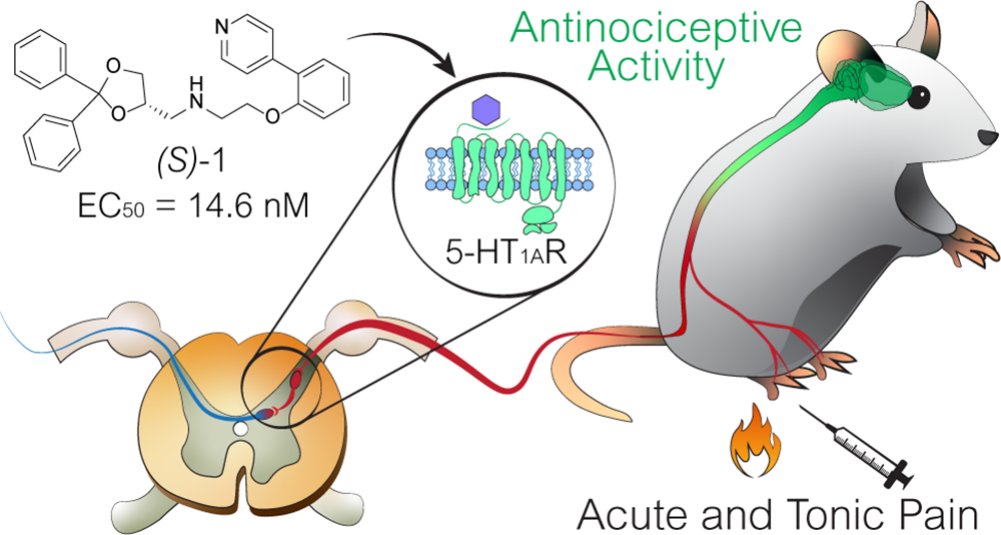

Opioids are the gold standard drugs for the treatment of acute and chronic severe pain, although their serious side effects constitute a big limitation. In the search for new and safer drugs, 5-HT_1A_R agonists are emerging as potential candidates in pain relief therapy. In this work, we evaluated the affinity and activity of enantiomers of the two newly synthesized, potent 5-HT_1A_R agonists *N*-[(2,2-diphenyl-1,3-dioxolan-4-yl)methyl]-2-[2-(pyridin-4-yl)phenoxy]ethan-1-ammonium hydrogenoxalate (*rac***-1**) and *N*-((2,2-diphenyl-1,3-dioxolan-4-yl)methyl)-2-(2-(1-methyl-1*H*-imidazol-5-yl)phenoxy)ethan-1-ammonium hydrogenoxalate (*rac***-2**) *in vitro* and *in vivo*. The role of chirality in the interaction with 5-HT_1A_R was evaluated by molecular docking. The activity of the *rac***-1** was tested in mouse models of acute pain (hot plate) and severe tonic nociceptive stimulation (intraplantar formalin test). *Rac***-1** was active in the formalin test with a reduction in paw licking in both phases at 10 mg/kg, and its effect was abolished by the selective 5-HT_1A_R antagonist, WAY-100635. The eutomer (*S*)-**1**, but not the racemate, was active during the hot plate test at 10 and 20 mg/kg, and this effect was abolished by 30 min treatment with WAY-100635 at 30 min. Similarly to 8-OH-DPAT, (*S*)-**1** evoked a slow outward current and depressed spontaneous glutamatergic transmission in superficial dorsal horn neurons, more effectively than *rac*-**1**. The eutomer (*S*)-**1** showed promising developability properties, such as high selectivity over 5-HT subtypes, no interaction with the μ receptors, and low hepato- and cardiotoxicity. Therefore, (*S*)-**1** may represent a potential candidate for the treatment of acute and chronic pain without having the adverse effects that are commonly associated with the classic opioid drugs.

## Introduction

Acute and chronic pain, both nociceptive and neuropathic, are involved in many illnesses and conditions, posing a great challenge to public health. Although opioids are widely used for the treatment of acute and chronic severe pain, their serious adverse effects, primarily respiratory depression, in addition to tolerance and drug addiction, represent a big limitation.^1^ Thus, there is still an urgent medical need for better therapeutic options. The serotoninergic system, originating in the brain stem and exerting a prevalent antinociceptive effect in the spinal cord, represents an innovative target for new analgesic compounds.^2^ All seven types of serotoninergic receptors (5-HTRs), including various isoforms, are present in the spinal cord, where they play complex roles in modulating pain.^3^ 5-HT_1A_Rs are highly expressed throughout the pain neuroaxis where they exert a clear antinociceptive action by inhibiting the transmission of nociceptive signals.^3^ Indeed, full and partial 5-HT_1A_R agonists have shown to be effective in the treatment of pain.^4−7^ In addition, the use of 5-HT_1A_ agonists in combination with opioids could be particularly advantageous, since they are able to reduce addiction, by decreasing the reward effects of opioids^8^ and to revert opioid-induced respiratory depression.^9^

5-HT2 receptors seem to exert opposite effects on pain, since the activation of the 5-HT_2A_ receptors located on rat spinal cord dorsal horn interneurons was shown to potentiate inflammatory pain and facilitate excitatory synaptic transmission.^10,11^ Furthermore, 5-HT_2_ receptor agonists mediate major side effects like anxiety, insomnia, sexual dysfunction, and dysfunction in platelet aggregation, as well as side effects related to the inhibition of dopamine signaling (e.g., Parkinsonian-like tremor, galactorrhea, cognitive dysfunction, extrapyramidal symptoms).^12−16^ 5-HT_2_ receptors are also involved in fibrosis in a variety of tissues like lung, kidney, heart, or liver, which is particularly important with chronic use of 5-HT_2_ receptor ligands or agents that inhibit serotonin reuptake.^17,18^ Thus, designing ligands featuring low 5-HT_2_ receptor affinity and higher selectivity at the 5-HT_1A_ subtype could reduce the occurrence of unwanted effects. 5-HT_6_ receptors are coupled to Gαs protein and are expressed in regions regulating pain processing, such as the cortex, thalamus, PAG, spinal cord, and dorsal root ganglia (DRG). They seem to exert a prevalent pronociceptive effect, in both formalin-evoked nociceptive behavior and animal models of neuropathic pain.^19−21^ 5-HT_7_ receptors are also located in several regions along the pain axis, in nociceptive DRG neurons and in subpopulations of the spinal cord dorsal horn. These receptors could exert pronociceptive activity, since 5-HT_7_ agonists increase pain behavior during the second phase of the formalin test, while antagonists reduce tactile allodynia induced by nerve injury. However, antinociceptive effects of 5-HT_7_ receptors have also been reported in several pain models.^3,22^ These data indicate that both 5-HT_6_ and 5-HT_7_ receptors are actively involved in nociception, with a possible prevalent pronociceptive action. For this reason, serotoninergic compounds with high selectivity for 5-HT_1A_ and low affinity for both 5-HT_6_ and 5-HT_7_ receptors could be candidates as antinociceptive agents.

**Figure 1:**
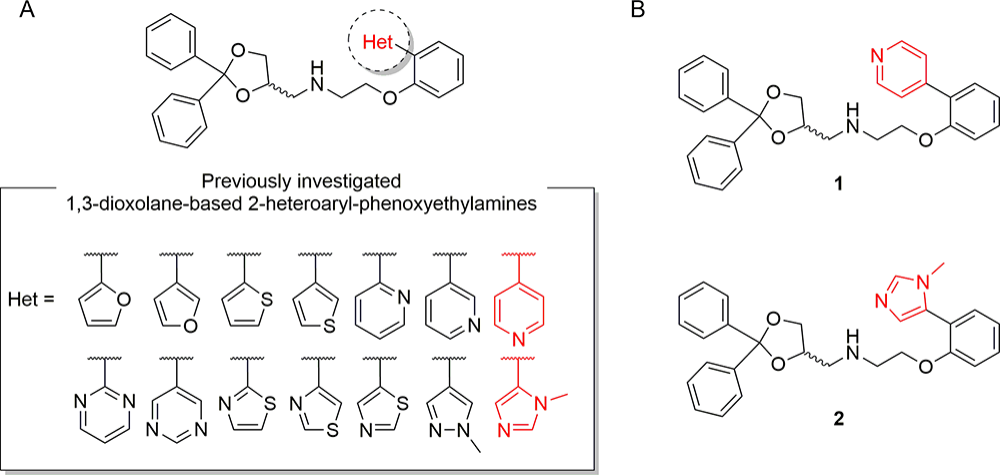
(A) General structure of the heteroaryl-phenoxyethylamines library. (B) Most promising compounds **1** and **2**.

Phenoxyethylamines are promising chemotypes for obtaining potent and selective 5-HT_1A_R agonists.^23−27^ In our previous work, a library of 2-heteroaryl-phenoxyethylamines was prepared, and the role of an electron-poor/rich heteroaryl moiety was investigated throughout the SAR studies.^28^ In that study, the 4-pyridinyl (**1**) and imidazolyl (**2**) derivatives emerged as the most promising compounds, showing excellent 5-HT_1A_R affinity (p*K*_i_ = 9.2 and 8.98, respectively) and selectivity (5-HT_1A_/α_1_ = 135 and 132, respectively) (Figure 1). The study of functional activity revealed that both compounds behaved as agonists, with **1** having the highest potency (pD_2_ = 8.83). Despite the quite low potency of **2** (pD_2_ = 5.46), this compound presented peculiar behavior: in its series, it was the only one to show efficacy above 100%, with a relative effectiveness (*E*_max_) of 240%. Therefore, **1** and **2** were selected for further investigation. Since both compounds present one chiral center, we first evaluated the pharmacological profile of each enantiomer, as a fundamental step in the drug development process. It is well-known that enantiomers of chiral drugs may exhibit different biological activity. Indeed, the disconnection between the high binding affinity and the low potency of **2**, in the GTPγS assay, might suggest that enantiomers exert opposite effects.

In this study, the enantiomeric pairs (*R*)-**1**/(*S*)-**1** and (*R*)-**2**/(*S*)-**2** were prepared using stereoselective synthesis and tested for binding affinity and functional activity at the molecular target 5-HT_1A_R, as well as for binding affinity to other subtypes (5-HT_2A_R, 5-HT_2C_R, 5-HT_6_R, and 5-HT_7_R) closer to the 5-HT_1A_. Molecular docking studies were performed to investigate the role of chirality in the interaction with the 5-HT_1A_R. The impact on μ opioid receptors, as well as the hepato- and cardiotoxicity, were also investigated *in vitro*. Considering the key role played by 5-HT_1A_R agonists in pain, the racemate **1** and the most potent enantiomer (*S*)-**1** were evaluated *in vivo* in two pain models to assess the antinociceptive potential. To confirm their efficacy in modulating spinal nociceptive circuitries through 5-HT_1A_R, *rac*-**1** and (*S*)-**1** were also tested *in vitro* by electrophysiological recordings from spinal cord dorsal horn neurons.

## Results and Discussion

### Chemistry

**Scheme 1:**
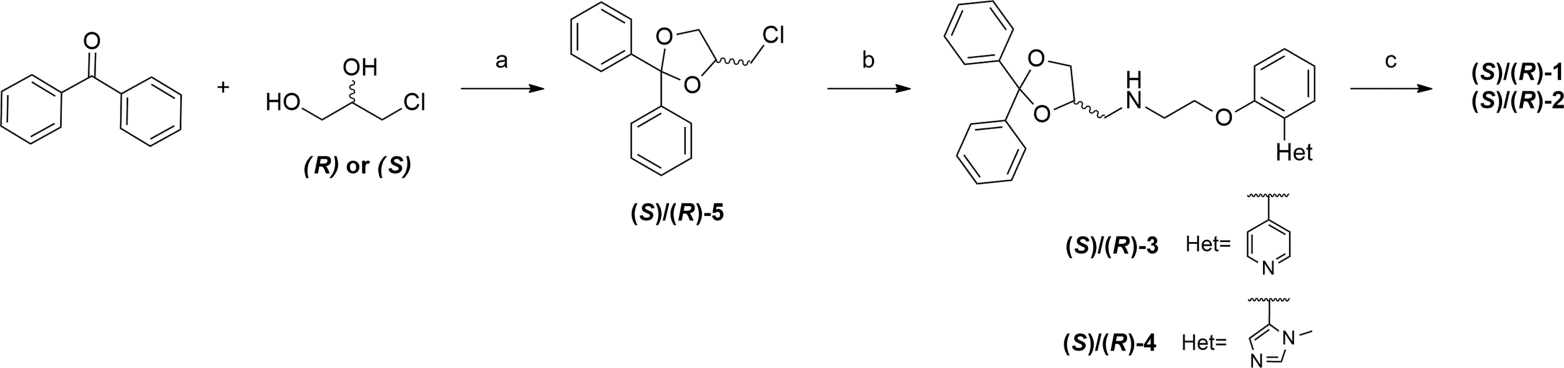
Reagents and Conditions: (a) benzophenone (1 equiv), (*R*)- or (*S*)-3-chloro-propan-1,2-diol (1.5 equiv), *p*TSA (0.1 equiv), dry toluene, refl., 18 h, 90–05% yield; (b) **6** or **7** (1.2 equiv), KI (cat.), DMSO, 90 °C, 24–48 h, yield: 47% (for (*S*)**-3**), 18% (for (*R*)**-3**), 21% (for (*S*)**-4**) and 36% (for (*R*)**-4**); (c) anhydrous oxalic acid (1.1 equiv), diethyl ether, r.t., 48 h, yield: 85% (for (*S*)**-1**), 75% (for (*R*)**-1**), 49% (for (*S*)-**2**), 27% (for (*R*)**-2**)

The tested compounds were synthesized as previously reported with slight modifications.^28^ (*S*)**-** and (*R*)**-1** and **2** were prepared as oxalate salts by reaction of the corresponding enantiopure free amines (*S*)/(*R*)**-3** and (*S*)/(*R*)**-4** with anhydrous oxalic acid, followed by crystallization from dry diethyl ether. The free amines (*S*)/(*R*)**-3** and (*S*)/(*R*)**-4** were directly obtained by standard S_N_2 reaction between the appropriate (*S*) or (*R*)-4-(chloromethyl)-2,2-diphenyl-1,3-dioxolane ((*S*) or (*R*)-**5**) and 2-(2-(pyridin-4-yl)phenoxy)ethan-1-amine (**6**) or 2-(2-(1-methyl-1*H*-imidazol-5-yl)phenoxy)ethan-1-amine (**7**). The S_N_2 reaction was performed in DMSO at 90 °C for 24–48 h, using potassium iodide as a catalyst (Scheme 1). The enantiomeric pure aliphatic chlorides (*S*) or (*R*)**-5** were prepared by condensation of benzophenone and the chiral (*S*)- or (*R*)-3-chloro-propan-1,2-diols, in refluxing toluene, using *p*-toluensulfonic acid (*p*TSA) as a catalyst and a Dean–Stark trap to remove the water formed. No racemization was observed, and the two aliphatic chlorides (*S*) and (*R*)**-5** were obtained.

**Scheme 2:**
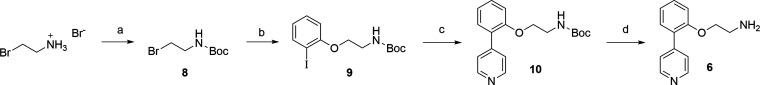
Reagents and Conditions: (a) Di-*tert*-butyl dicarbonate (1.1 equiv), THF/aq. NaHCO_3_ sat. sol. 1:1, r.t., 6 h, quant. yield; (b) 2-iodo-phenol (0.9 equiv), K_2_CO_3_ (2.5 equiv), DMF, 60 °C, 2 h, 97% yield; (c) 4-pyridyl boronic acid (1.5 equiv), 2N K_2_CO_3_ (2.5 equiv), tetrakis(triphenylphosphine)palladium(0) (0.03 equiv), dioxane, 80 °C, 18 h, 72% yield; (d) TFA/DCM 1:1, 0 °C to r.t., 3 h, yield: 95%

The two 2-heteroarylphenoxyethylamines **6** and **7** were prepared by slightly improving the previously reported synthetic approach.^29^ As shown in Scheme 2, the amino group of 2-bromoethylamine hydrobromide was protected by reacting with di-*tert*-butyl dicarbonate in THF/saturated NaHCO_3_ aqueous solution, providing *N*-Boc-2-bromoethylamine (**8**) in quantitative yield. **8** was then reacted with 2-iodophenol in standard S_N_2 conditions, obtaining *N*-Boc-(2-iodophenoxy)ethylamine (**9**). The synthesis of the two biaryl scaffolds was performed using two different methods. The 4-phenylpyridine scaffold was obtained by reaction of **9** via Suzuki cross-coupling with the commercially available 4-pyridylboronic acid, in dioxane using tetrakis(triphenylphosphine)palladium(0) as a catalyst and 2N K_2_CO_3_ aqueous solution as a base, to give **10** in 72% yield.

**Scheme 3:**
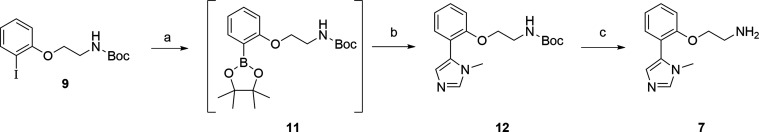
Reagents and Conditions: (a) Bis(pinacolato)diboron (1.5 equiv), PdCl_2_(dppf) (0.05 equiv), AcOK (3 equiv), DMSO, 80 °C, overnight; (b) 5-bromo-1-methyl-imidazole (1 equiv), 2N K_2_CO_3_ (2.5 equiv), tetrakis(triphenylphosphine)palladium(0) (0.1 equiv), dioxane, 80 °C, 24 h, 71% yield; (c) TFA/DCM 1:1, 0 °C to r.t., 2 h, yield: 89%

Since the 1-methylimidazole-5-boronic acid was not commercially available, the synthesis of the 1-methyl-5-phenyl-1*H*-imidazole scaffold was performed by conversion of **9** into the corresponding phenylboronic acid pinacol ester (**11**) by Miyaura borylation reaction. In order to avoid the loss of product due to decomposition on silica gel, **11** was not purified and the crude extract was used directly in the next step. Suzuki cross-coupling of **11** with 5-bromo-1-methylimidazole afforded **12** in 71% yield over the two synthetic steps. Lastly, the *N*-Boc cleavage of **10** and **12** under acid conditions (TFA/DCM 1:1) provided the final amines **6** and **7** (Scheme 3).

#### Competition Binding and Functional Studies at 5-HT_1A_R

The two enantiomeric pairs (*R*)-**1**/(*S*)-**1** and (*R*)-**2**/(*S*)-**2** and the corresponding racemates **1** and **2** were tested for their binding affinity and functional activity at the main target 5-HT_1A_R. The results are shown in Table 1. As revealed by one-way ANOVA, all the compounds showed high, nanomolar affinities, p*K*_i_ ranging from 7.92 to 8.39. Moreover, the compound pairs did not differ in their binding affinity for these receptor subtypes, thus highlighting lack of stereoselectivity, as previously reported by us for a 1,3-dioxolane-based compound.^24^

**Table 1:** Binding Affinity (p*K*_i_), Agonist Potency (pEC_50_), and Relative Effectiveness (*E*_max_) of the Enantiomeric Pairs (*S*)-**1**/(*R*)-**1** and (*S*)-**2**/(*R*)-**2** and Racemates at the 5-HT_1A_R

	5-HT_1A_^a^	5-HT_1A_^b^,^c^	
compound	p*K*_i_ ± SEM	pEC_50_ ± SEM	*E*_max_ (%) ± SEM
rac-1	8.04 ± 0.05	7.0 ± 0.2	151 ± 4.2
(*S*)-**1**	8.03 ± 0.10	7.8 ± 0.12*	138 ± 2.9
(*R*)-**1**	8.35 ± 0.9	7.2 ± 0.18	158 ± 5.9
rac-2	8.13 ± 0.10	7.4 ± 0.09	184 ± 2.7##
(*S*)-**2**	8.39 ± 0.11	7.6 ± 0.11*	175 ± 2.7##,***
(*R*)-**2**	7.92 ± 0.07	7.2 ± 0.09	143 ± 3.6
buspirone	7.52 ± 0.04	-	-
8-OH-DPAT	-	7.5 ± 0.11	152 ± 2.4
serotonin	-	6.8 ± 0.08	192 ± 4.9

a HEK293 cells transfected with human 5-HT_1A_R. The results are expressed as means ± SEM from at least two independent experiments.

b Rat hippocampal membranes. The results are expressed as the means ± SEM of three independent experiments. Basal 5-HT_1A_R activation was set to 100%.

c ***p < 0.001 and *p < 0.05 vs the (*R*) enantiomer; ##*p* < 0.01, and vs 8-OH-DPAT.

Functional activity at 5-HT_1A_R was evaluated in rat hippocampal membrane preparations. As shown in Table 1, all optical isomers and racemates stimulated G-protein activation with nanomolar affinity, pEC_50_ ranging from 7.0 to 7.8. One-way ANOVA revealed differences in binding affinities within each compound pair and when compared with 8-OH-DPAT (*F*_3,11_ = 7.76, *p* < 0.01 for pair **1** and *F*_3,11_ = 6.8, *p* < 0.05 for pair **2**). Unexpectedly, (*R*)-**2** and (*S*)-**2** enantiomers did not show an opposite contribution to the functional readout, both acting as agonists. Noteworthy, G-protein activation elicited by the four optical isomers, as well as their racemates, involved 5-HT_1A_R receptors. As seen in Table SI-1, WAY-100635 shifted the agonist-stimulated [^35^S]GTPγS binding curve to the right without affecting the maximal response. In addition, WAY-100635 completely abolished agonist-induced stimulation, implying a fully 5-HT_1A_-dependent effect (Table SI-2).

In detail, (*S*)**-2** and *rac***-2** acted as full agonists and expressed efficacy superior to 8-OH-DPAT (*p* < 0.01), while (*S*)**-1**, *rac***-1**, (*R*)**-1**, and (*R*)**-2** were partial agonists as their stimulation of [^35^S]GTPγS binding was half (or lower) as efficacious as for serotonin. Interestingly, both *S* optical isomers of each couple were more potent than the *R* pairs (*p* < 0.05), with a eudismic ratio (EC_50_*S*/*R*) of 4.0 and 2.6 for **1** and **2**, respectively. Promisingly, both *S* optical isomers showed an pEC_50_ value comparable ((*S*)**-2**) or higher ((*S*)**-1**) than the reference agonist, 8-OH-DPAT. Overall, the functional binding analysis carried out on the new enantiomeric pairs highlighted a slight degree of enantioselectivity for the *S* enantiomers in terms of 5-HT_1A_R activation.

#### Molecular Modeling of 5-HT_1A_ Receptor and Docking Studies

To get a better understanding of the role of chirality on the interaction with the biological target, molecular docking studies were performed at the binding site of the human 5-HT_1A_R protein. In the absence of the crystallographic structure for 5-HT_1A_R, before proceeding with the docking calculation, our previous ligand-based homology model was refined by using the recently available X-ray crystallographic structure of the human 5-HT_1B_R (PDBID = 5V54; resolution = 3.9 Å).^30^ As shown by the alignment of the 5-HT_1A_R primary sequence with that of the template, a consistent number of residues proved to be conserved between these two receptor subtypes (Figure SI-1). Accordingly, the modeled 5-HT_1A_ backbone conformation featured good correspondence with that of the GPCR template (RMSD = 0.854 Å) (Figure SI-2 and SI-3). Superimposition of the theoretical model of the 5-HT_1A_ receptor onto the template allowed us to identify the putative binding site of compounds targeting 5-HT_1A_R, based on the corresponding binding site of methiothepin. A number of studies describe a unique receptor cavity involved in the binding with 5-HT_1A_R full agonists, partial agonists, and antagonists.^31,32^ In particular, H-bond interactions between agonists and Asp116 and Asn386 were suggested, falling in a crevice delimited by Phe112, Ile113, Asp116, Lys191, while partial agonists, as well as antagonists, were H-bonded at least with Asp116. In agreement with the literature, the putative binding mode of the antagonist methiothepin displayed one salt-bridge with the key residue Asp116 and cation−π interactions with Phe112. The tricyclic core detected hydrophobic contacts with Ile189, Ala203, Trp358, Phe361, and Phe362 (Figure SI-4).

**Figure 2:**
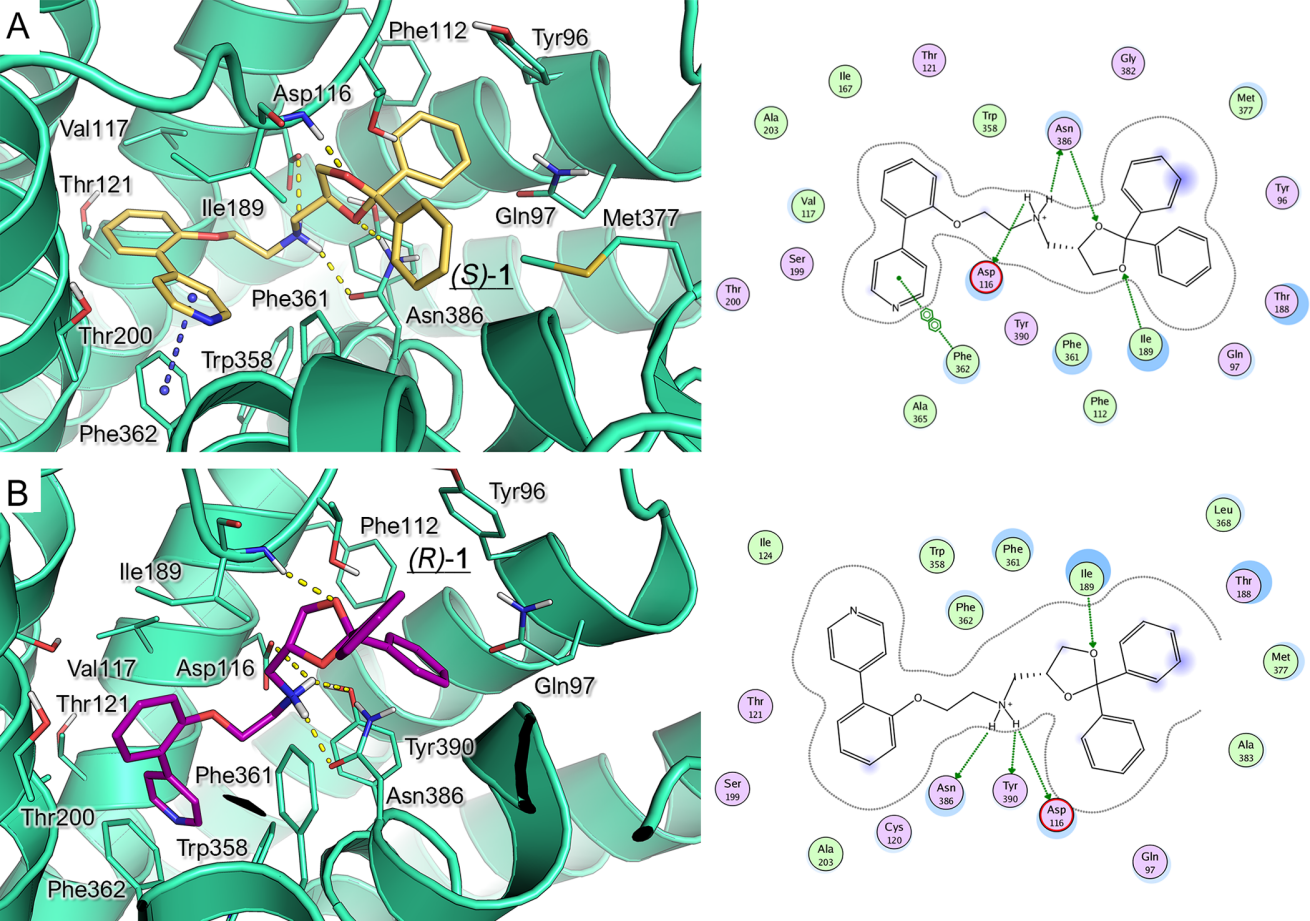
Binding mode of (*S*)**-1** (**A**, yellow carbon) and (*R*)**-1** (**B**, magenta carbon) within the modeled 5-HT_1A_R (green diagram). H-bonds and π–π stacking are represented as yellow and blue dashed lines, respectively. The derived ligplots are shown on the right; the polar and hydrophobic residues are colored in magenta and green, respectively.

Then, (*S*)**-1** and (*R*)**-1** enantiomers were analyzed. According to our calculations, they feature the same positioning within the protein cavity. Both optical isomers displayed the key salt-bridge with Asp116 and one H-bond with Asn386, through the protonated nitrogen atom of the amine chain. The diphenyl-dioxolane ring was located in proximity to Tyr96, Phe112, and Tyr390, featuring π–π stacking and van der Waals contacts, while one of the two oxygen atoms of the dioxolane was H-bonded to Ile189 (Figure 2). The biaryl moiety moved toward a hydrophobic pocket delimited by Val117, Thr121, Ile167, Ile189, Thr200, Trp358, Phe361, Phe362, and Ala365. This allowed the agonist to be better stabilized at the protein crevice, exhibiting additional π–π and hydrophobic contacts. While (*S*)**-1** featured a further H-bond with Asn386 thanks to the dioxolane substituent, the (*R*)**-1** was properly oriented within the receptor crevice by means of additional polar contacts involving the protonated basic group and the Tyr390 side-chain. Remarkably, this kind of interaction was featured by the (*S*)**-2** dioxolane ring, described as follows, exhibiting comparable binding affinity.

**Figure 3:**
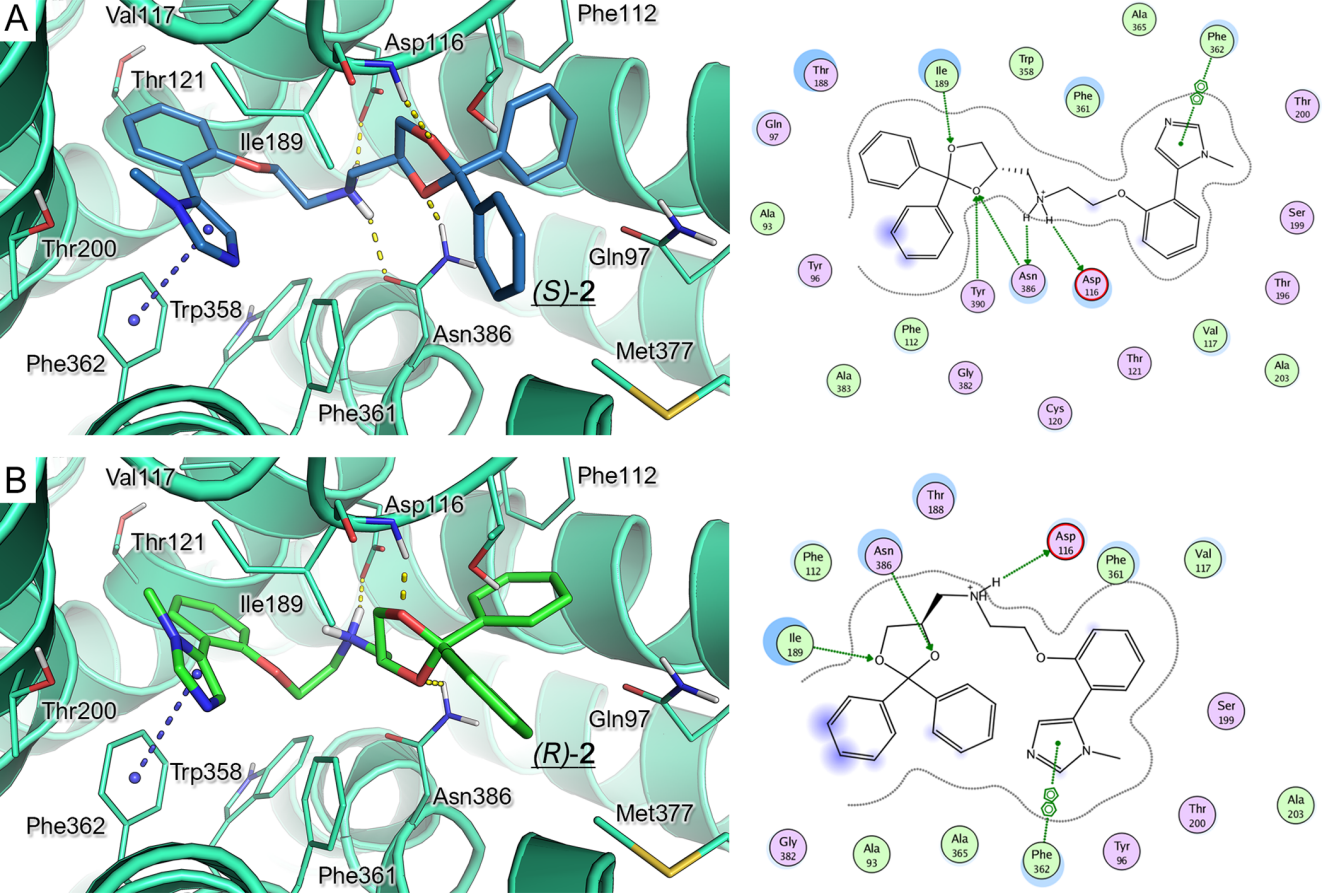
Binding mode of (*S*)-**2** (**A**, yellow carbon) and (*R*)-**2** (**B**, pink carbon) within the modeled 5-HT_1A_R (green diagram). The H-bonds and π–π stacking are represented as yellow and blue dashed lines, respectively. The derived ligplots are shown on the right; the polar and hydrophobic residues are colored in pink and green, respectively.

The (*S*)**-2** and (*R*)**-2** enantiomers were also analyzed. As described for (*S*)**-1**, the (*S*)**-2** enantiomer experienced a similar docking pose (Figure 3A), being endowed with numerous hydrophobic interactions with the receptor, by means of the imidazole ring with respect to the (*R*)**-2** enantiomer (Figure 3B). Once again, (*S*)**-2**, but not (*R*)**-2**, displayed a further H-bond with Asn386, via the amino group, while one of the two oxygen atoms of the dioxolane core was engaged in polar contacts with Tyr390. These findings are in agreement with the binding affinity data at 5-HT_1A_R, with (*S*)**-2** being 3-fold more potent than (*R*)**-2**.

Taken together, these results revealed the relevant role played by the hydrogen bond between the amino group and the Asn386 residue and/or Tyr390, contributing to the stabilization of the (*R*)*-***1** and (*S*)*-***2** optical isomers.

### Developability Studies

#### Competition Binding Studies at 5-HT_2A_, 5-HT_2C_, 5-HT_6_, and 5-HT_7_ (Off-Targets)

The two enantiomeric pairs (*R*)-**1**/(*S*)-**1** and (*R*)-**2**/(*S*)-**2** and the corresponding racemates **1** and **2** were tested for their binding affinity at the 5-HT subtypes (5-HT_2A_, 5-HT_2C_, 5-HT_6_, and 5-HT_7_) that are structurally and evolutionally closest to the 5-HT_1A_R. Affinity at the 5-HT_2A_R and 5-HT_2C_R subtypes was studied in rat frontal cortex membrane preparations, whereas affinity at 5-HT_6_R and 5-HT_7_R was performed in stably transfected HEK293 cells. The results are reported in Table 2. All the compounds showed weaker binding affinity at both 5-HT_2_R subtypes compared with 5-HT_1A_R. As revealed using one-way ANOVA, all the compounds bound to 5-HT_2A_R with relatively low affinity (*F*_3,11_ = 433.1, *p* < 0.001 for pair **1** and *F*_3,11_ = 201, *p* < 0.001 for pair **2**). All the compounds also showed considerably lower affinity to 5-HT_2A_R (p*K*_i_ ranging from 5.80 to 6.38), when compared with the selective antagonist, ketanserin (p*K*_i_ = 8.27). (*S*)**-1** showed almost 2-fold improved affinity compared with (*R*)*-***1** (p*K*_i_ S/R = 1.99, *p* < 0.05). Conversely, no stereoselectivity was observed for the (*R*) and (*S*) enantiomers of compound **2**.

**Table 2:** Binding Affinity (p*K*_i_) of the Enantiomeric Pairs (*S*)-**1**/(*R*)-**1** and (*S*)-**2**/(*R*)-**2** and Racemates at the 5-HT_2A_R, 5-HT_2C_R, 5-HT_6_R, and 5-HT_7_R

	5-HT_2A_^a^	5-HT_2C_^a^	5-HT_6_^b^	5-HT_7_^b^
compound	p*K*_i_ ± SEM	p*K*_i_ ± SEM	p*K*_i_ ± SEM	p*K*_i_ ± SEM
*rac*-**1**	5.95 ± 0.054	6.44 ± 0.21	5.12 ± 0.05	6.29 ± 0.08
(*S*)-**1**	6.10 ± 0.055*	6.02 ± 0.12	5.49 ± 0.03	6.40 ± 0.02
(*R*)-**1**	5.80 ± 0.056	6.04 ± 0.12	5.37 ± 0.05	6.30 ± 0.03
*rac*-**2**	6.38 ± 0.12	6.48 ± 0.18	5.30 ± 0.04	6.18 ± 0.04
(*S*)-**2**	6.00 ± 0.044)	6.50 ± 0.32*	5.26 ± 0.02	6.22 ± 0.06
(*R*)-**2**	6.05 ± 0.056	5.90 ± 0.14)	5.30 ± 0.05	6.10 ± 0.03
ketanserin	8.27 ± 0.06	-	-	-
RS-102221	-	8.34 ± 0.12	-	-
olanzapine	-	-	7.91 ± 0.05 (	-
clozapine	-	-	-	7.21 ± 0.02

a Rat frontal cortex membranes. The results are expressed as means ± SEM of three independent experiments.

b HEK293 cells transfected with human 5-HT_1A_R. The results are expressed as means ± SEM from at least two independent experiments. *p < 0.05 vs the (*R*) enantiomer.

One-way ANOVA revealed that all the compounds also showed low binding affinity at the 5-HT_2C_R (p*K*_i_ ranging from 5.9 to 6.48), compared with the selective 5-HT_2C_R antagonist, RS-102221 (p*K*_i_ = 8.34) (*F*_3,11_ = 6.7, *p* < 0.05 for pair **1** and *F*_3,11_ = 4.5, *p* < 0.05 for pair **2**). No stereoselectivity was observed for compound **1** at the 5-HT_2C_R; however, the (*S*)**-2** enantiomer showed preference for the 5-HT_2C_R over the (*R*)**-2** enantiomer (p*K*_i_ S/R = 3.98). On the other hand, all the compounds showed low, micromolar or submicromolar affinity at the 5-HT_6_R and 5-HT_7_R, respectively.

Interestingly, all the compounds showed favorable selectivity profiles toward the 5-HT_1A_R (Table 3).

**Table 3:** Selectivity Profile of the Enantiomeric Pairs (*S*)-1/(*R*)-1 and (*S*)-2/(*R*)-2 and the Racemates at the 5-HT_1A_ vs 5-HT_2A_, 5-HT_2C_, 5-HT_6_, and 5-HT_7_ Receptors

selectivity^a^				
compound	5-HT_2A_/5-HT_1A_	5-HT_2_/5-HT_1A_	5-HT_6_/5-HT_1A_	5-HT_7_/5-HT_1A_
*rac*-**1**	123	40	832	56
(*S*)-**1**	85	102	347	43
(*R*)-**1**	355	204	955	112
*rac*-**2**	56	45	676	89
(*S*)-**2**	245	78	1349	148
(*R*)-**2**	74	105	417	66

a Selectivity ratio was calculated as an Antilog [p*K*_i_ (5-HT_1A_R) – p*K*_i_ (5-HT_2A_R or 5-HT_2C_R or 5-HT_6_R or 5-HT_7_R)].

Notably, the affinity profile revealed a difference within the same enantiomeric pair, (*R*)-**1** and (*S*)-**2** showing the highest selectivity profile. These compounds might represent useful pharmacological tools to study the role of 5-HT_1A_R subtype in different biological systems.

#### Cell Viability and Toxicity Studies

**Figure 4:**
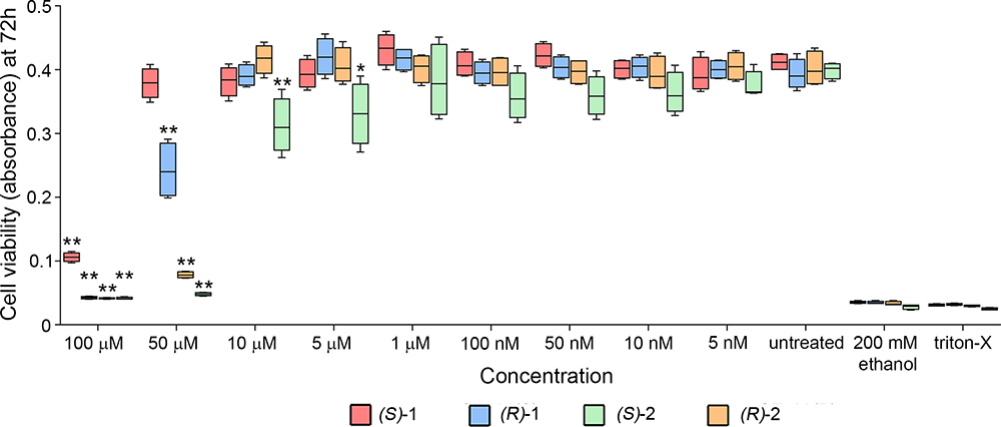
Impact on liver hepatocellular carcinoma Hep-G2 cell viability of (*S*)-**1**, (*R*)-**1**, (*S*)-**2**, and (*R*)-**2**. Cells were maintained 72 h in the presence of increasing concentrations of the compounds and viability was assessed by MTT assay. 200 mM ethanol and 5% Triton X-100 treated cells served as controls and data were shown by the box and whiskers plot. Two-way ANOVA and Dunnett’s post-test, *p* < 0.001*, *p* < 0.0001**, *n* = 4, versus untreated samples.

Hepatotoxicity and cardiotoxicity are two of the major causes of failure during drug development. *In vitro* cell-based viability assay using the Hep-G2 cell line of hepatic origin was used as a tool for safety evaluation in the early stages of drug discovery. The impact of nanomolar–micromolar concentrations of compounds (*S*)-**1**, (*R*)-**1**, (*S*)-**2**, and (*R*)-**2** on hepatic cell viability was evaluated by 3-(4,5-dimethylthiazol-2-yl)-2,5-diphenyltetrazolium bromide (MTT) assay after 72 h of treatment (Figure 4). Compounds (*S*)-**1**, (*R*)-**1**, and (*R*)-**2** showed very low toxicity at concentrations ≤10 μM, while (*S*)-**2** decreased cell viability at 10 and 5 μM (two-way ANOVA, *p* < 0.001, *n* = 4). At 50 μM, (*R*)-**1**, (*S*)-**2**, and (*R*)-**2** negatively impacted cell viability compared with untreated (control cells), while (*S*)-**1** had no effect (two-way ANOVA, *p* < 0.0001, *n* = 4). Therefore, compound (*S*)**-1** had lowest hepatotoxicity.

#### CiPA hERG QPatch Assay

Given the best functional potency and the lower impact on hepatic cell viability of (*S*)-**1**, this compound was profiled for activities on the human voltage gated potassium ion channel hERG, using the QPatch electrophysiological platform. The results showed that (*S*)-**1** does not inhibit hERG at the concentrations of 0.1 and 1 μM, indicating that this compound is suitable for drug development (Table 4).

**Table 4:** Mean % Inhibition of the Maximum Tail Current of (*S*)-**1** and the Reference Compound E-4031 in hERG-CHO Automated Patch-Clamp

	% inhibition of tail current in hERG-CHO		
compound	10 μM	1 μM	0.1 μM
(*S*)-**1**	66.65	5.61	–3.35
E-4031	-	-	83.14

To exclude any interaction with the opioid receptors, (*S*)-**1** was tested *in vitro* on mouse spinal cord slices, in the presence of naloxone, a nonselective and competitive opioid receptor antagonist. These data are presented in the section regarding the electrophysiological studies (Figure 11).

Based on the high potency, good safety profile, and favorable ADME properties (including the ability of **1** to permeate, by passive diffusion, MDCK-MDR1 monolayers, mimicking the BBB, indicating high brain uptake, and low efflux ratio^28^), the racemate **1** and its eutomer (*S*)-**1** were selected for *in vivo* studies.

### *In Vivo* Studies

#### Assessment of the Antinociceptive Activity in the Formalin Test

The formalin test was chosen as a tonic pain model for the assessment of potential analgesic activity of compound **1** in mice. Intraplantar administration of formalin (5%, 10 μL) produces a biphasic nocifensive behavioral response (i.e., biting or licking the injected hind paw). The acute nociceptive phase, reflecting the chemical activation of sensory C-fibers, lasts for the first 10 min, while the second inflammatory phase takes place between 15 and 50 min and corresponds with the development of nociceptive sensitization in the dorsal horn of the spinal cord.^33^

**Figure 5:**
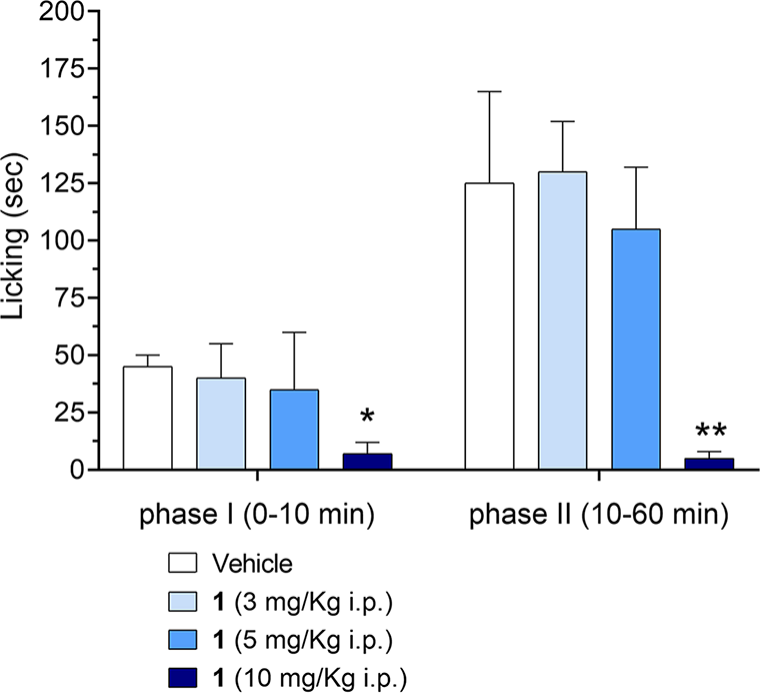
Effect of intraperitoneal (i.p.) injection of **1** (3, 5, or 10 mg/kg) on the first (0–10 min) and second (10–60 min) phases of the formalin test. The test compound or vehicle was injected 15 min before the intraplantar injection of formalin. The data are expressed as the means ± SEM of 8–10 mice per group. *p < 0.05 vs the respective groups of mice injected with the vehicle.

As shown in Figure 5, compound **1** was administered 15 min before formalin, at doses of 3, 5, and 10 mg/kg i.p. The 10 mg/kg dose was able to induce significant analgesic effects during the first and second phases of the formalin test (*p < 0.05). Morphine (10 mg/kg, i.p.) was used as a positive control and essentially eliminated the response to formalin in both phases. Pretreatment with the selective 5-HT_1A_R antagonist WAY-100635 (3 mg/kg, i.p.), 30 min before the administration of **1** (10 mg/kg i.p.), prevented its analgesic effect during the second phase (^**#**^*p* < 0.05), confirming the activation of the 5-HT_1A_ receptor.

**Figure 6:**
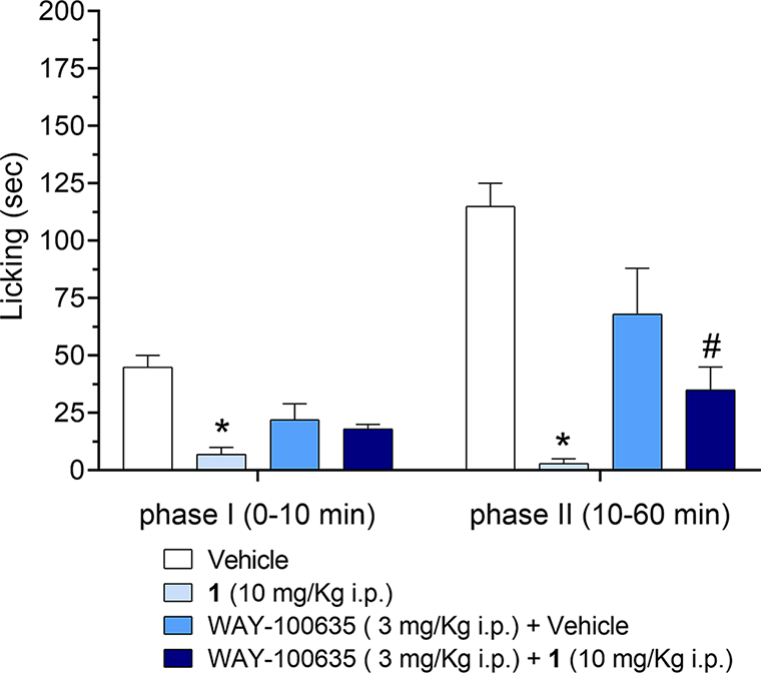
Effect of WAY-100635 (3 mg/kg, i.p.) on analgesia induced by **1** (10 mg/kg, i.p.) during the first (0–10 min) and second (10–60 min) phases of the formalin test. The test compound or vehicle was injected 15 min prior to the intraplantar injection of formalin. WAY-100635 was injected 30 min prior to **1** or vehicle. The data are expressed as the means ± SEM of 8–10 mice per group. *p < 0.05 vs the respective groups of mice injected with the vehicle. #*p* < 0.05 vs mice treated with **1** (10 mg/kg, i.p.).

WAY-100635 (3 mg/kg, i.p.), *per se*, at least at the dose used, did not alter the licking response after injection with formalin (Figure 6). These data show that **1** was effective in decreasing the acute activation of nociceptive fibers (first phase) and the inflammatory response, leading to a facilitated state of the nociceptive system (second phase). These results are consistent with the previously described antinociceptive action of 5-HT_1A_R agonists in the formalin pain model.^34,35^ A discrepancy between the analgesic effect of 5-HT_1A_R agonists in both phases and the blockade by the antagonist WAY-100635, only during the second phase, has also been observed in previous studies.^36^ This discordance could be due to the different involvement of 5-HT_1A_R in acute pain and central pain sensitization.

#### Assessment of Analgesic Activity in the Hot Plate Test

**Figure 7:**
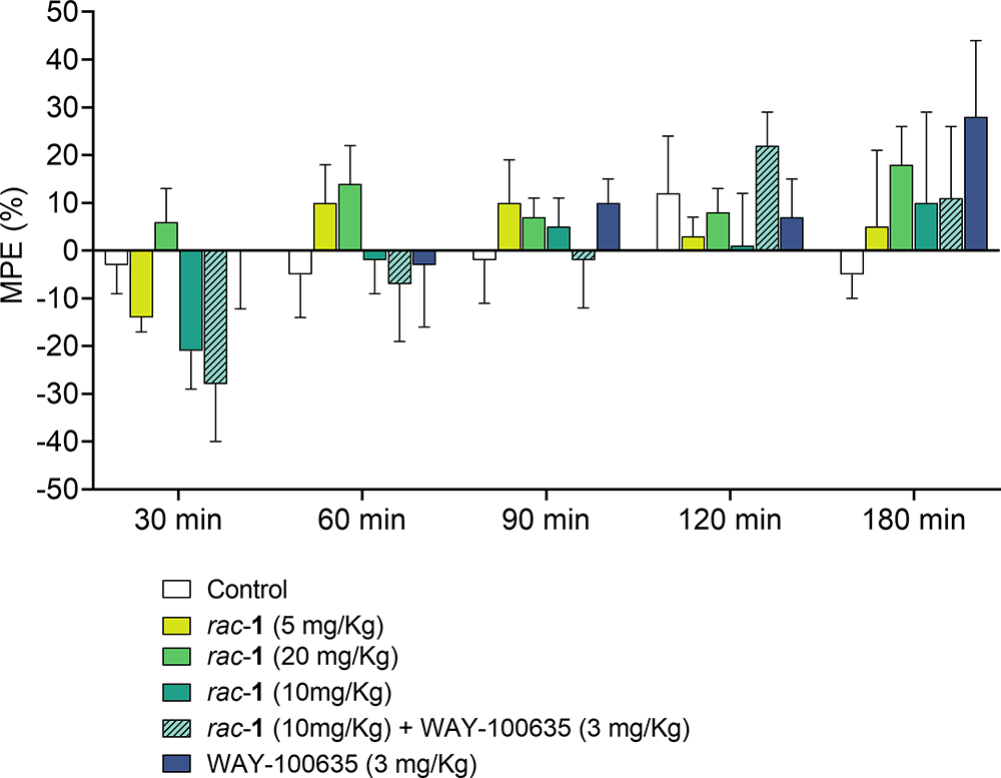
Influence of *rac*-**1** on nociceptive reactions assessed using the hot plate test in mice. Nociceptive reactions were measured over the total period of 180 min (30, 60, 90, 120, and 180 min after compound administration). The data are expressed as the mean ± SEM values.

The antinociceptive properties of **1** were also assessed using the hot plate test, an acute, thermally induced pain model (Figure 7). This procedure involves a supraspinal reflex mediated by opioid receptors.^37,38^ The paws of mice are extremely sensitive to heat at temperatures that do not cause damage to their skin. The animals respond by jumping, withdrawing, or licking their paws. The response time can be increased by the administration of central acting analgesics. Drugs like acetylsalicylic acid or metamizole type, with peripheral action, do not have an effect on these responses. The hot plate test has been used by many researchers and has been deemed suitable for the evaluation of analgesics that act centrally but not peripherally.^39^

The hot plate test was performed before and after intraperitoneal administration of the compound **1** in mice at three different doses (5, 10, and 20 mg/kg). Statistical analysis of the results obtained did not reveal any significant change in the MPE values at the chosen time intervals at all tested doses. At 30 min, administration of the *rac***-1** at 5 and 10 mg/kg seemed to have a pronociceptive action. However, this effect was not statistically significant, nor did it seem to be attributable to 5-HT_1A_R activation, being not inhibited by the 5-HT_1A_ antagonist WAY-100635.

**Figure 8:**
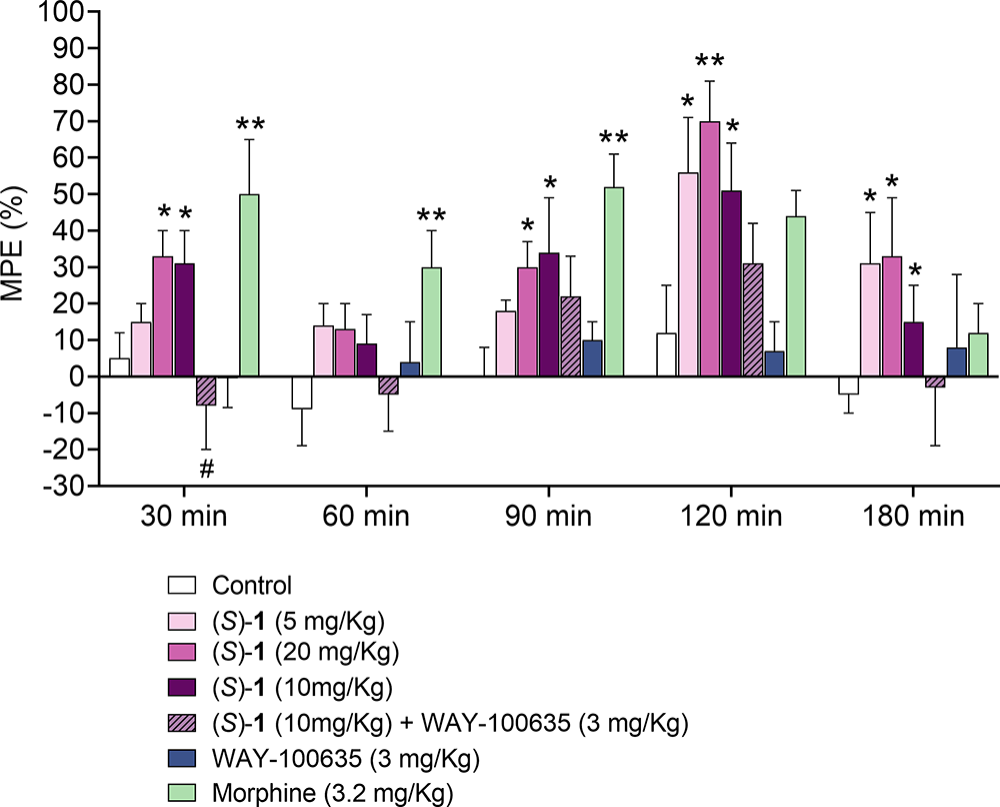
Influence of (*S*)-**1** enantiomer on nociceptive reactions assessed using the hot plate test in mice. Nociceptive reactions were measured over the total period of 180 min (30, 60, 90, 120, and 180 min after compound administration). The data are expressed as the mean ± SEM values.

Since in the functional experiments the (*S*)*-***1** enantiomer showed 6-fold higher activity compared with the racemate **1**, the hot plate test was also performed on (*S*)-**1**. As shown in Figure 8, one-way ANOVA showed significant changes in the MPE values at 30 (*F*_5,37_ = 3.952; *p* < 0.01), 90 (*F*_5,36_ = 2.081; *p* < 0.05), 120 (*F*_5,34_ = 4.084; *p* < 0.01), and 180 min (*F*_5,34_ = 1.763; *p* = 1.1471) after the injection for (*S*)-**1**. This compound, at the dose of 20 mg/kg, significantly increased the reaction time to the thermal stimulus at 30 (*p* < 0.05), 90 (*p* < 0.05), 120 (*p* < 0.01), and 180 min (*p* < 0.05). At the dose of 10 mg/kg, the reaction time to the thermal stimulus was significantly increased at 30, 90, 120, and 180 (*p* < 0.05) min, while at 5 mg/kg a significant effect was only noted at 120 and 180 min (*p* < 0.05). The selective 5-HT_1A_R antagonist WAY-100635 (3 mg/kg) significantly reversed the antinociceptive effect of the (*S*)-**1** enantiomer at the dose of 10 mg/kg at 30 min (*p* < 0.05). Morphine was employed as a positive control at the dose of 3.2 mg/kg.

The behavioral data show that racemate **1** has an antinociceptive action in the formalin test, while it is not effective in the hot plate test, where only the (*S*)-**1** enantiomer has significant activity. This discrepancy could be due to a higher agonist sensitivity of spinal versus supraspinal 5-HT_1A_R, as previously reported.^5^

### *In Vitro* Electrophysiological Studies

#### Effects of rac-**1**, (*S*)**-1**, and 8-OH-DPAT on Activity of Spinal Cord Dorsal Horn Neurons

**Figure 9:**
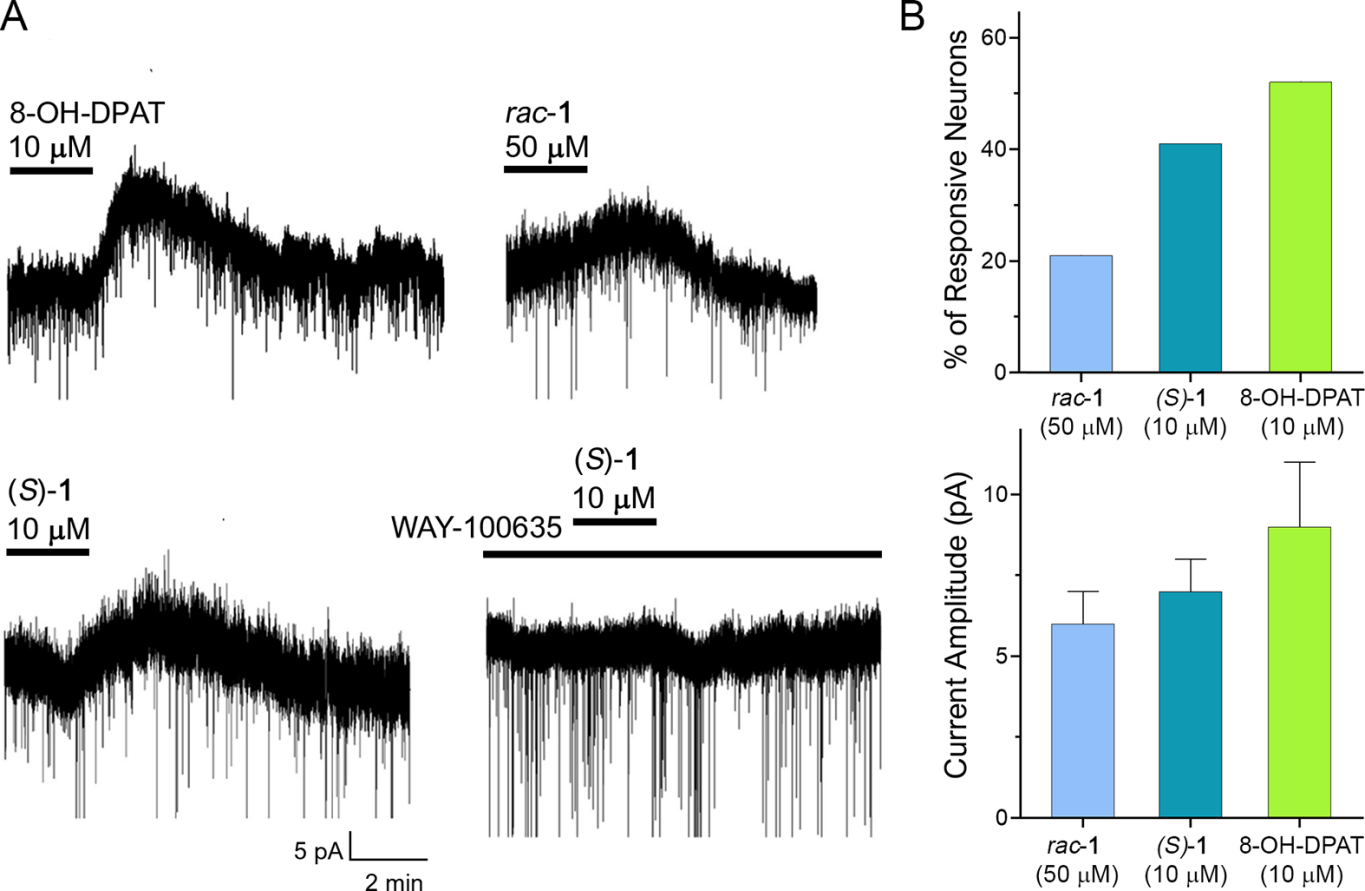
(A) Representative traces of the outward currents observed after 2 min of the compound application. Outward currents evoked by *rac***-1** and (*S*)-**1** had similar kinetics to those generated by 8-OH-DPAT. Application of WAY-100635 (10 μM) blocked the effect of (*S*)-**1** in all 7 neurons tested. (B) Top: (*S*)-**1** (10 μM) was effective in evoking outward currents in a higher percentage of neurons (5 out of 12) than *rac*-**1** 50 μM (5 out of 23). 8-OH-DPAT generated an outward current in 6 out of 11 cells. Neurons were considered responsive if they showed an outward current distinguishable from the baseline noise (>3 pA). Bottom: Mean amplitudes of the outward currents evoked by the tested compounds in dorsal horn neurons, showing no significant differences among the 3 groups (one-way ANOVA, *P* = 0.53).

*Rac*-**1** and its more active enantiomer (*S*)**-1** were also tested *in vitro* by electrophysiological recordings from spinal cord dorsal horn neurons. Recordings were obtained from a total of 65 superficial dorsal horn neurons. At the beginning of the experiment, the resting potential was measured: neurons showing a value more positive than −50 mV were discarded. Lamina I–II neurons were then recorded in voltage clamp at −60 mV. Bath application of the 5-HT_1A_ agonist 8-OH-DPAT (10 μM) evoked a slow outward current in 6 out of the 11 tested neurons (Figure 9A). This current, carried by potassium ions, corresponds with neuron hyperpolarization and inhibition of neuron excitability, as previously described in most lamina I–II neurons.^3,40,41^ The *rac*-**1** induced outward currents of similar amplitudes in 5 out of 23 neurons when applied at 50 μM, while the concentration of 10 μM was not effective. Its *S* enantiomer exerted a more potent effect, evoking an outward current, at 10 μM, in 5 out of 12 tested neurons. The current amplitudes and kinetics were similar to those obtained by applying 10 μM 8-OH-DPAT (Figure 9B). In the presence of the 5-HT_1A_R antagonist WAY-100635 (10 μM), (*S*)**-1** failed to evoke the outward currents in all 7 neurons tested, showing that the compound activates 5-HT_1A_R on the recorded dorsal horn neurons.

**Figure 10:**
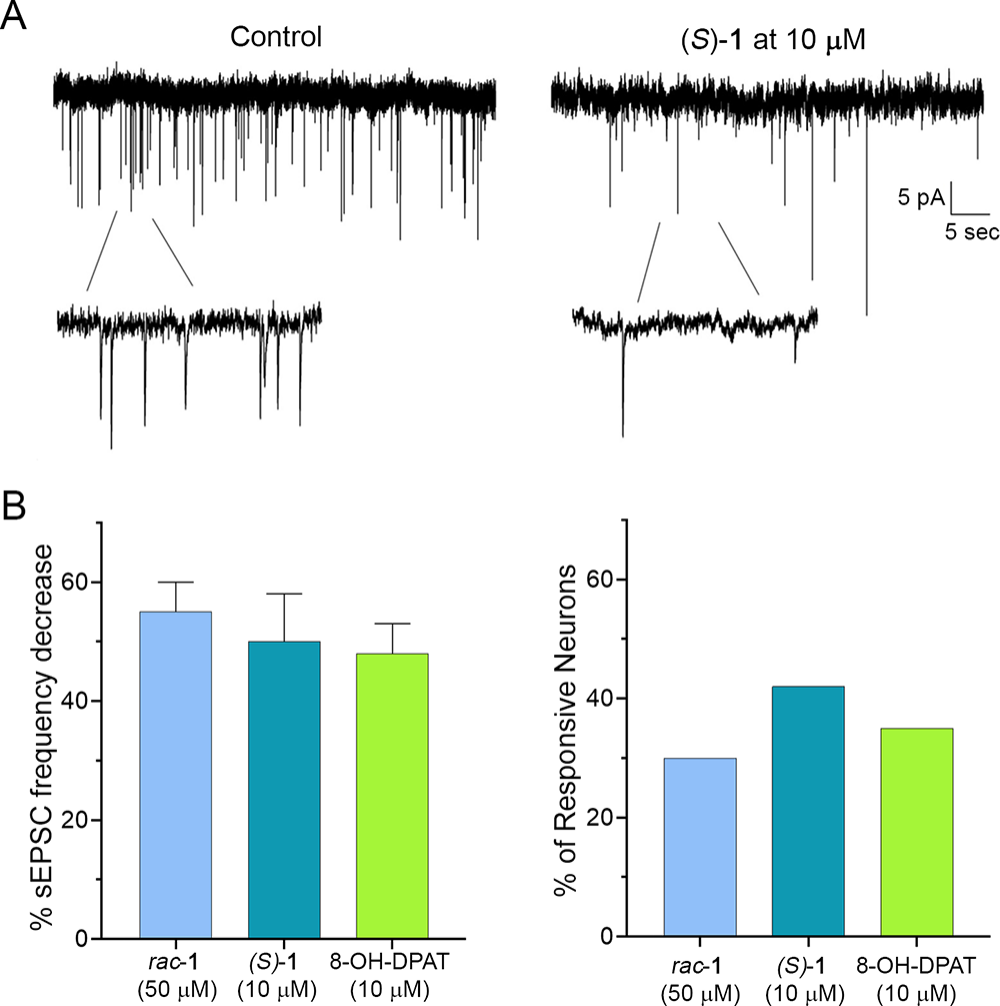
(A) Left: Representative traces of spontaneous excitatory postsynaptic currents (sEPSCs) recorded from superficial dorsal horn neurons at −60 mV. Right: Application of (*S*)-**1** (10 μM) caused a significant decrease of sEPSC frequency. In this particular neuron, (*S*)-**1** did not evoke any slow outward current. (B) Left: (*S*)-**1** (10 μM) caused a similar sEPSC frequency decrease as *rac*-**1** (50 μM) and 8-OH-DPAT (10 μM) (one-way ANOVA, *P* = 0.80). Right: sEPSC frequency decrease was observed in a higher percentage of neurons for (*S*)-**1** (5 out of 12) than for *rac*-**1** (7 out of 23). 8-OH-DPAT was effective in 4 out of 11 tested neurons.

As shown in Figure 10A, fast inward currents, corresponding with spontaneous excitatory postsynaptic currents (sEPSCs) mediated by glutamate, were also visible in voltage-clamp recordings. During the application of *rac*-**1** (50 μM) or (*S*)**-1** (10 μM), the frequency of the synaptic currents significantly decreased in a subpopulation of neurons (Figure 10). A decrease of EPSC frequency was also produced by 10 μM 8-OH-DPAT, as previously reported in the superficial dorsal horn.^11,42^ The enantiomer (*S*)-**1** was more potent than the *rac*-**1**, producing comparable effects at a lower concentration (10 vs 50 μM). This indicates that, similarly to 8-OH-DPAT, *rac*-**1** and (*S*)-**1** can inhibit glutamate release from presynaptic neurons and fiber terminals, by acting on 5-HT_1A_R.

In summary, the data from the electrophysiological experiments show that the more potent enantiomer (*S*)-**1** activates 5-HT_1A_R in the mouse superficial dorsal horn and is effective in inhibiting nociceptive transmission, by decreasing neuronal excitability and glutamate release.

A 5-fold concentration (50 vs 10 mM) was required for *rac*-**1** to produce similar effects on dorsal horn neurons compared to its *S* enantiomer. Interestingly, a similar ratio was observed between the EC_50_ values determined for the two compounds (92.1 and 14.6 nM, respectively).

#### Effects of (*S*)-**1** on Dorsal Horn Neuron in the Presence of Naloxone

**Figure 11:**
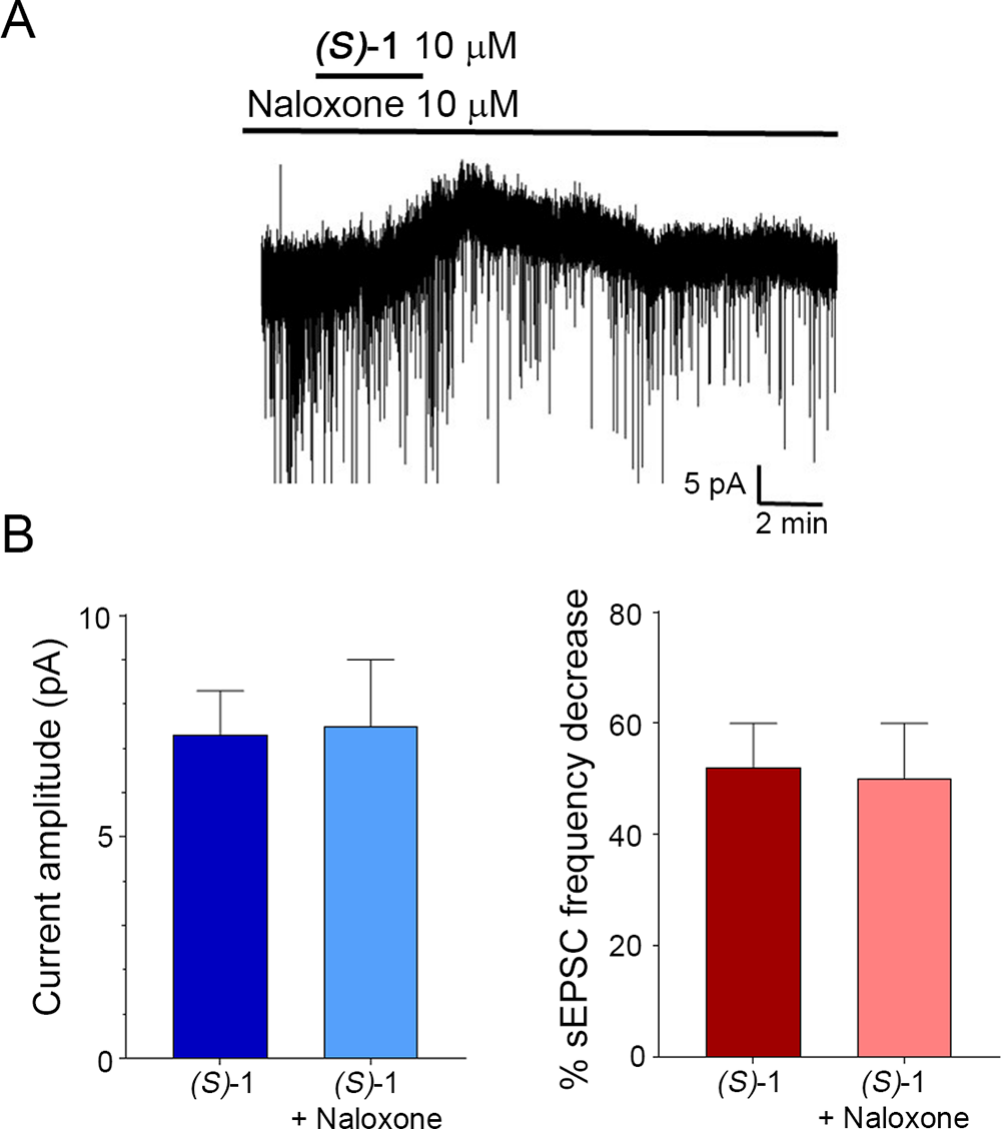
Effects of the enantiomer (*S*)-**1** on spinal cord dorsal horn neurons, in the presence of the opioid receptor antagonist naloxone. A: Representative recording, showing the appearance of a slow outward current, superimposed by a decrease of the spontaneous excitatory postsynaptic currents (sEPSCs). B: Amplitudes of outward currents (left) and percentage sEPSC frequency decreases (right), produced by (*S*)-**1**, in the absence or presence of naloxone. No significant differences were detected for both outward currents and sEPSC frequency (*t* test, *P* = 0.86 and *P* = 0.88, respectively).

To exclude any interaction with the opioid receptors, the more active compound (*S*)-**1** was tested *in vitro* on mouse spinal cord slices, in the presence of naloxone, a nonselective and competitive opioid receptor antagonist. As shown in Figure 11, application of 10 μM (*S*)-**1** still caused an outward current in 5 out of 12 neurons and a significant decrease in sEPSC frequency in 6 out of 12 neurons. The recorded cells were located in superficial dorsal horn (laminae I and II outer), where opioid receptors (particularly μ receptors) are abundantly expressed at both the pre- and post-synaptic sites.^43−45^ The amplitude of the outward current and the percentage decrease in sEPSC frequency were not significantly different from those observed in the absence of naloxone, indicating that the effects of the enantiomer (*S*)-**1** on dorsal horn neurons are not due to the activation of μ receptors. The lack of activity on μ opioid receptors is fundamental for the development of (*S*)-**1** as an alternative non-opioid analgesic drug.

## Conclusions

The enantiomeric pairs (*S*)**/**(*R*)**-1** and (*S*)**/**(*R*)**-2** were synthesized and tested for their affinity and functional activity at the main target 5-HT_1A_R. They were all selective for 5-HT_1A_R, acting as full or partial agonists. The compound pairs did not differ in their binding affinity for 5-HT_1A_R, thus highlighting a lack of stereoselectivity, as confirmed by the docking calculations. However, a slight degree of enantioselectivity was seen for both *S* optical isomers with respect to 5-HT_1A_R activation. *In vivo* experiments showed that the racemate **1** exerts a potent analgesic action at both peripheral and spinal levels, as highlighted by the effect of this compound in the formalin test. At the supraspinal level, the enantiomer (*S*)**-1** was able to produce an analgesic effect, as shown in the hot plate test. In both pain models, the effects were antagonized by WAY-100635, confirming the involvement of the 5-HT_1A_R. In addition, the results obtained in the *in vitro* spinal cord dorsal horn preparation confirmed that (*S*)**-1** is more effective than the racemate at inhibiting neuron excitability. Moreover, (*S*)-**1** exhibited a good developability profile, not interacting with the μ opioid receptors and showing low hepato- and cardiotoxicity. Taken together, our data suggest that (*S*)**-1** is a valid alternative to opioids in the treatment of acute and chronic pain.

## Methods

### Chemistry

All the reagents and solvents were commercially available from Sigma-Aldrich. The moisture-sensitive reactions were performed under an inert atmosphere of nitrogen. The following solvents have been abbreviated: diethyl ether (Et_2_O), dichloromethane (DCM), dimethylformamide (DMF), dimethyl sulfoxide (DMSO), chloroform (CHCl_3_), cyclohexane (Cy), ethyl acetate (EtOAc), methanol (MeOH). Each reaction was monitored by TLC on Merck 60G F^254^ plates and detected at 254 nm. All the compounds, unless otherwise specified, were purified by flash column chromatography using silica gel 60 (230–400 mesh, ASTM) supplied by Merck. The melting points were determined with Stuart SMP3 apparatus and are uncorrected. NMR spectra were recorded on a Bruker 400 spectrometer with ^1^H at 400.134 MHz and ^13^C at 100.62 MHz. Chemical shifts were referenced to the solvent residual peaks. ^1^H NMR peak patterns are as follows: s (singlet), d (doublet), t (triplet), m (multiplet), br (broad singlet). The purity of the compounds was determined by elemental analysis (C, H, N), which was performed on a Carlo Erba 1106 Analyzer and the results shown here are within ±0.4% of the theoretical values. Optical rotation (λ) was measured with a Polarimeter 240C (cell-length 100 mm, volume 1 mL) from PerkinElmer (Milan, Italy).

#### General Procedure for the Synthesis of the Oxalate Salts (*S*)/(*R*)-**1** and (*S*)/(*R*)-**2**

To a solution of the appropriate amine (*S*)-**3**, (*R*)-**3**, (*S*)-**4**, or (*R*)-**4** (1 equiv) in 5 mL of anhydrous diethyl ether at room temperature and under nitrogen atmosphere, anhydrous oxalic acid (1.2 equiv) was added. The suspension was stirred for 30 min and left to settle for 48 h. The precipitate was collected by filtration, washed with anhydrous diethyl ether, and dried to afford the title compound.

##### (*S*)- or (*R*)-*N*-[(2,2-Diphenyl-1,3-dioxolan-4-yl)methyl]-2-[2-(pyridin-4-yl)phenoxy]ethan-1-ammonium hydrogenoxalate, (*S*)/(*R*)-**1**

(*S*)-**1** (0.050 g, 0.092 mmol, 85% yield) as a white solid, m.p 162–164 °C. ^1^H NMR (400 MHz, DMSO-*d*_6_) δ 2.95 (dd, *J* = 8.16, 12.68 Hz, 1H), 3.02–3.06 (m, 1H), 3.27–3.29 (m, 2H), 3.64 (dd, *J* = 6.52, 8.32 Hz, 1H), 4.01 (dd, *J* = 7.00, 8.32 Hz, 1H), 4.24–4.27 (m, 2H), 4.29–4.34 (m, 1H), 7.13 (t, *J* = 7.52 Hz, 1H), 7.19 (d, *J* = 7.96 Hz, 1H), 7.26–7.45 (m, 12H), 7.54 (d, *J* = 6.12 Hz, 2H), 8.54 (d, *J* = 6.12 Hz, 2H). ^13^C NMR (101 MHz, DMSO-*d*_6_) δ 46.77, 50.05, 65.23, 67.28, 73.13, 109.70, 113.14, 121.67, 124.08, 125.73, 125.81, 127.20, 128.02, 128.16, 128.26, 130.34, 130.44, 141.76, 141.87, 145.36, 149.33, 154.90, 163.61. Elemental analysis (CHN) calculated for C_31_H_30_N_2_O_7_: C: 68.62; H: 5.57; N: 5.16. Found: C: 68.42; H: 5.70; N: 4.99.

(*R*)-**1** (0.045 g, 0.082 mmol, 75% yield), as a white solid, mp 165–166 °C. ^1^H NMR (400 MHz, DMSO-*d*_6_) and ^13^C NMR (101 MHz, DMSO-*d*_6_) identical to that of (*S*)-**1**. Elemental analysis (CHN) calculated for C_31_H_30_N_2_O_7_: C: 68.62; H: 5.57; N: 5.16. Found: C: 68.48; H: 5.62; N: 5.06.

##### (*S*)- or (*R*)-*N*-((2,2-Diphenyl-1,3-dioxolan-4-yl)methyl)-2-(2-(1-methyl-1*H*-imidazol-5-yl)phenoxy)ethan-1-ammonium hydrogenoxalate, (*S*)/(*R*)-**2**

(*S*)-**2** (0.041 g, 0.075 mmol, 49% yield), as a pale yellow solid, mp 102–103 °C. ^1^H NMR (400 MHz, DMSO-*d*_6_) δ 2.91 (dd, *J* = 8.76, 12.9 Hz, 1H), 3.00 (dd, *J* = 3.36, 12.9 Hz, 1H), 3.30–3.37 (m, 2H), 3.47 (s, 3H), 3.68 (dd, *J* = 6.54, 8.46 Hz, 1H), 4.03 (dd, *J* = 7.08, 8.46 Hz, 1H), 4.28 (t, *J* = 5.34 Hz, 2H), 4.28–4.33 (m, 1H), 6.98 (s, 1H), 7.10 (t, *J* = 6.0 Hz, 1H), 7.17 (d, *J* = 8.28 Hz, 1H), 7.29–7.35 (m, 5H), 7.38–7.48 (m, 7H), 7.85 (s, 1H). ^13^C NMR (101 MHz, DMSO-*d*_6_) δ 32.06, 46.35, 49.77, 64.87, 67.25, 72.74, 109.83, 112.61, 118.11, 121.31, 125.78, 125.92, 126.69, 128.05, 128.22, 128.24, 128.31, 129.39, 130.41, 131.82, 138.26, 141.67, 141.77, 155.55, 163.04. Elemental analysis (CHN) calculated for C_30_H_31_N_3_O_7_: C: 66.04; H: 5.73; N: 7.70. Found: C: 65.82; H: 5.98; N: 7.49.

(*R*)-**2** (0.035 g, 0.064 mmol, 27% yield), as a yellow solid, 104–105 °C. ^1^H NMR (400 MHz, DMSO-*d*_6_) and ^13^C NMR (101 MHz, DMSO-*d*_6_) identical to that of (*S*)-**2**. Elemental analysis (CHN) calculated for C_30_H_31_N_3_O_7_: C: 66.04; H: 5.73; N: 7.70. Found: C: 65.92; H: 5.87; N: 7.69.

#### General Procedure for the Synthesis of Amines (*S*)/(*R*)-**3** and (*S*)/(*R*)-**4**

To a solution of (*R*)-**5** or (*S*)-**5** (1 equiv) in DMSO (3 mL) a solution of the appropriate amine **6** or **7** (1.2 equiv) in DMSO (2 mL) and KI (cat.) were added. The solution was stirred at 90 °C for 24–48 h and quenched with water. The aqueous layer was alkalinized at pH 14 with 1 N NaOH and extracted with EtOAc. The organic layer was washed with water, brine, dried over anhydrous Na_2_SO_4_, and concentrated. The crude extract was chromatographed over silica gel using EtOAc:MeOH 9:1 as the mobile phase.

##### (*S*)- or (*R*)-*N*-[(2,2-Diphenyl-1,3-dioxolan-4-yl)methyl]-2-[2-(pyridin-3-yl)phenoxy]ethanamine, (*R*)/(*S*)-**3**

(*S*)-**3** (240 mg, 0.53 mmol, 48% yield), as a yellow oil. ^1^H NMR (400 MHz, Chloroform-*d*) δ 1.79 (bs, 1H), 2.75 (dd, *J* = 4.4, 12.2 Hz, 1H), 2.84 (dd, *J* = 7.1, 12.2 Hz, 1H), 2.85–3.01 (m, 2H), 3.78 (dd, *J* = 6.4, 7.9 Hz, 1H), 3.91–4.19 (m, 3H), 4.27 (qd, *J* = 7.1, 4.2 Hz, 1H), 7.01 (dd, *J* = 1.0, 8.3 Hz, 1H), 7.08 (td, *J* = 1.0, 7.5 Hz, 1H), 7.17–7.39 (m, 8H), 7.39–7.51 (m, 6H), 8.56 (d, *J* = 3.9 Hz, 2H). ^13^C NMR (101 MHz, Chloroform-*d*): δ 48.69, 52.28, 68.06, 68.09, 76.19, 109.25, 112.75, 121.41, 124.30, 126.16, 126.28, 128.07, 128.16, 128.20, 130.15, 130.54, 142.20, 142.36, 146.38, 149.49, 155.75. [α]_20_^D^ = −10.9° (*c* = 0.01, CHCl_3_). Elemental analysis (CHN) calculated for C_29_H_28_N_2_O_3_: C: 76.97; H: 6.24; N: 6.19. Found: C: 76.92; H: 6.20; N: 6.26.

(*R*)-**3** (89 mg, 0.19 mmol, 18% yield) as a yellow oil. ^1^H NMR (400 MHz, Chloroform-*d*) and ^13^C NMR (101 MHz, Chloroform-*d*) identical to that of (*S*)-**3**. [α]_20_^D^ = +11.3° (*c* = 0.005, CHCl_3_).

##### (*S*)- or (*R*)-*N*-((2,2-diphenyl-1,3-dioxolan-4-yl)methyl)-2-(2-(1-methyl-1*H*-imidazol-5-yl)phenoxy)ethan-1-amine, (*S*)/(*R*)-**4**

(*S*)-**4** (222 mg, 0.48 mmol, 36% yield), as an amber oil. ^1^H NMR (400 MHz, Chloroform-*d*) δ 2.13 (bs, 1H), 2.79 (m, 2H), 2.98 (t, *J* = 5.16 Hz, 2H), 3.46 (s, 3H), 3.83 (dd, *J* = 7.8, 6.4 Hz, 1H), 4.08 (m, 3H), 4.30 (qd, *J* = 6.7, 4.5 Hz, 1H), 7.02 (m, 2H), 7.07 (m, 1H), 7.21–7.60 (m, 13H). ^13^C NMR (101 MHz, Chloroform-*d*): δ 32.04, 48.67, 52.24, 67.97, 68.35, 76.14, 109.93, 112.52, 119.30, 121.14, 126.20, 126.33, 128.06, 128.18, 128.22, 128.45, 130.21, 132.29, 138.30, 142.18, 142.36, 156.60. [α]_20_^D^ = −15.3° (*c* = 0.01, CHCl_3_). Elemental analysis (CHN) calculated for C_28_H_29_N_3_O_3_: C: 73.82; H: 6.42; N: 9.22. Found: C: 73.75; H: 6.49; N: 9.30.

(*R*)-**4** (169 mg, 0.37 mmol, 21% yield), as a yellow oil. ^1^H NMR (400 MHz, Chloroform-*d*) and ^13^C NMR (101 MHz, Chloroform-*d*) identical to that of (*S*)-**4**. [α]_20_^D^ = +16.5° (*c* = 0.01, CHCl_3_).

#### Synthesis of (*S*)- or (*R*)-4-(chloromethyl)-2,2-diphenyl-1,3-dioxolane (**5**)

The synthesis of the titled compounds was performed as previously reported, using (*S*)-3-chloro-propan-1,2-diol or (*R*)-3-chloropropan-1,2-diol to prepare (*S*)-**5** and (*R*)-**5**, respectively. ^1^H NMR (400 MHz, Chloroform-d) δ 3.40 (dd, *J* = 8.0, 10.9 Hz, 1H), 3.61 (dd, *J* = 4.7, 10.9 Hz, 1H), 3.95 (dd, *J* = 5.1, 8.6 Hz, 1H), 4.05 (dd, *J* = 6.6, 8.6 Hz, 1H), 4.36 (ddt, *J* = 4.9, 6.6, 8.1 Hz, 1H), 7.18–7.34 (m, 6H), 7.34–7.52 (m, 4H). [α]_20_^D^ = −43.6° (*c* = 0.03, CHCl_3_) for (*S*)-**5** and [α]_20_^D^ = +42.9° (*c* = 0.03, CHCl_3_) for (*R*)-**5**.

#### Synthesis of 2-(2-(Pyridin-4-yl)phenoxy)ethan-1-amine (**6**)

**8** (440 mg, 1.40 mmol, 1 equiv) was solubilized in a 1:1 mixture of TFA:DCM at 0 °C. The solution was stirred at room temperature for 3 h and concentrated. The residue was solubilized in water and the pH adjusted to 14 with pellets of NaOH. The aqueous phase was extracted with EtOAc and the organic layer washed with brine, dried over anhydrous Na_2_SO_4_, and concentrated. The titled compound was obtained as a pale yellow liquid (290 mg, 97% yield), which was pure enough to be used in the next step without further purification.

^1^H NMR (400 MHz,Chloroform-*d*) δ 1.71 (br s, 2H), 2.99 (t, *J* = 5.0 Hz, 2H), 3.99 (t, *J* = 5.0 Hz, 2H), 6.99 (d, *J* = 8.3 Hz, 1H), 6.91–6.99 (m, 1H), 7.27–7.42 (m, 2H), 7.39–7.47(m, 2H), 8.51–8.60 (m, 2H); ^13^C NMR (101 MHz, Chloroform-*d*): δ 52.48, 68.62, 112.57, 121.23, 124.14, 127.79, 129.90, 130.62, 146.18, 149.41, 153.67.

#### Synthesis of 2-(2-(1-Methyl-1*H*-imidazol-5-yl)phenoxy)ethan-1-amine (**7**)

The N-Boc cleavage of **9** was performed as reported above for the synthesis of **6**. Dark yellow liquid (89% yield). ^1^H NMR (400 MHz, Chloroform-*d*) δ 1.37 (bs, 2H), 2.98 (t, *J* = 5.3 Hz, 2H), 3.55 (s, 3H), 4.00 (t, *J* = 5.3 Hz, 2H), 6.95–7.18 (m, 3H), 7.21–7.32 (m, 1H), 7.37–7.47 (m, 1H), 7.53 (s, 1H). ^13^C NMR (101 MHz, Chloroform-*d*) δ 32.18, 41.42, 70.93, 112.67, 130.20, 132.18, 138.34, 156.68.

#### Synthesis of *tert*-Butyl (2-bromoethyl)carbamate (**8**)

To a stirred suspension of 2-bromoethylamine hydrobromide (1.0 g, 4.88 mmol, 1 equiv) in THF/aq. NaHCO_3_ saturated solution 1:1 (20 mL), di-*tert*-butyl decarbonate (1.17 g, 5.40 mmol, 1.1 equiv) was added. The mixture was stirred at room temperature for 6 h. The organic phase was evaporated, and the aqueous residue was extracted with Et_2_O. The organic layer was washed with brine, dried over anhydrous Na_2_SO_4_, and concentrated to give the titled compound as colorless liquid (1.09 g, quant. yield) that was pure enough to be used in the next step without further purification.

^1^H NMR (400 MHz, Chloroform-*d*) δ 1.44 (s, 9H), 3.44 (t, *J* = 5.9 Hz, 2H), 3.51 (t, *J* = 5.9 Hz, 2H), 4.94 (s, 1H). ^13^C NMR (101 MHz, Chloroform-*d*) δ 146.6, 85.0, 42.3, 28.2, 27.2.

#### Synthesis of *tert*-Butyl (2-(2-iodophenoxy)ethyl)carbamate (**9**)

To a solution of 2-iodophenol (1.18 g, 5.35 mmol, 1.2 equiv) in DMF (5 mL), K_2_CO_3_ (1.53 g, 11.15 mmol, 2.5 equiv) was added. The mixture was stirred at room temperature for 30 min and **8** (1.0 g, 4.46 mmol, 1 equiv) was added in one portion. The suspension was heated to 60 °C for 2 h and concentrated. The residue was resuspended in water and extracted with EtOAc. The organic layer was washed with Na_2_CO_3_ saturated solution, brine, dried over anhydrous Na_2_SO_4_, and concentrated. The crude was chromatographed over silica gel (mobile phase Cy:EtOAc 8:2) to give 1.57 g (97% yield) of a pale yellow liquid.

^1^H NMR (400 MHz, Chloroform-*d*) δ 1.47 (s, 9H), 3.60–3.61 (m, 2H), 4.08 (t, *J* = 4.8 Hz, 2H), 5.14 (bs, 1H), 6.75 (t, *J* = 8.0 Hz, 1H), 6.82 (d, *J* = 8.0 Hz, 1H), 7.30 (t, *J* = 8.0 Hz, 1H), 7.78 (d, *J* = 8.0 Hz, 2H). ^13^C NMR (101 MHz, Chloroform-*d*) δ 28.4, 40.0, 68.6, 79.5, 86.8, 112.5, 123.0, 129.6, 139.4, 155.9, 157.0.

#### Synthesis of *tert*-Butyl (2-(2-(pyridin-4-yl)phenoxy)ethyl)carbamate (**10**)

To a solution of **9** (726 mg, 2 mmol, 1 equiv) in dry dioxane (15 mL) at room temperature and under argon atmosphere, 4-pyridylboronic acid (369 mg, 3 mmol, 1.5 equiv) and the 2 N K_2_CO_3_ aqueous solution (3 mL, 6 mmol, 2 equiv) were added. The mixture was degassed with argon-vacuum cycles before the addition of tetrakis(triphenylphosphine)palladium(0) (70 mg, 0.06 mmol, 0.03 equiv). The reaction was heated to 80 °C under vigorous stirring for 18 h and concentrated. The residue was partitioned between EtOAc and Na_2_CO_3_ saturated solution. The organic phase was collected, washed with brine, dried over anhydrous Na_2_SO_4_, and concentrated. The crude was chromatographed over silica gel (mobile phase EtOAc 100%) to give 480 mg (72% yield) of a yellow liquid.

^1^H NMR (400 MHz, Chloroform-*d*) δ 1.41 (s, 9H), 3.44 (q, *J* = 5.2 Hz, 2H), 4.03 (t, *J* = 5.2 Hz, 2H), 4.72 (bs, 1H), 6.98 (d, *J* = 8.2 Hz, 1H), 7.06 (t, *J* = 8.2 Hz, 1H), 7.31–7.34 (m, 2H), 7.44 (d, *J* = 4.8 Hz, 1H), 8.62 (d, *J* = 6.0 Hz, 2H).

#### Synthesis of *tert*-Butyl (2-(2-(1-methyl-1*H*-imidazol-5-yl)phenoxy)ethyl)carbamate (**12**)

To a solution of **9** (500 mg, 1.38 mmol, 1 equiv) in DMSO (5 mL), bis(pinacolato)diboron (530 mg, 2.07 mmol, 1.5 equiv), PdCl_2_(dppf) (51 mg, 0.07 mmol, 0.05 equiv), and potassium acetate (405 mg, 4.14 mmol, 3 equiv) were added and stirred in a closed vessel at 80 °C, overnight. The mixture was diluted with water and extracted with Et_2_O. The organic layer was dried over anhydrous Na_2_SO_4_ and concentrated to give a sticky white liquid (**11**) which was used directly in the next Suzuki cross-coupling reaction. 5-Bromo-1-methylimidazole (221 mg, 1.38 mmol, 1 equiv) was solubilized in dry dioxane (20 mL) at room temperature and under argon atmosphere. The crude of the previous Miyaura reaction (**11**) and the 2N K_2_CO_3_ aqueous solution (0.7 mL, 1.38 mmol, 1 equiv) were added. The mixture was degassed with argon-vacuum cycles before the addition of tetrakis(triphenylphosphine)palladium(0) (159 mg, 0.138 mmol, 0.1 equiv). The reaction was heated to 80 °C under vigorous stirring for 24 h and concentrated. The residue was partitioned between EtOAc and Na_2_CO_3_ saturated solution. The organic phase was collected, washed with brine, dried over anhydrous Na_2_SO_4_, and concentrated. The crude was chromatographed over silica gel (mobile phase EtOAc:MeOH 95:5) to give 311 mg (71% yield) of a yellow liquid.

^1^H NMR (400 MHz, Chloroform-*d*) δ 1.42 (s, 9H), 3.40 (m, 2H), 3.51 (s, 3H), 4.03 (t, *J* = 5.2 Hz), 4.84 (bs, 1H), 6.89–7.07 (m, 3H), 7.20–7. 32 (m, 1H), 7.33–7.42 (m, 1H), 7.50 (s, 1H). ^13^C NMR (101 MHz, Chloroform-*d*) δ 28.51, 32.43, 40.38, 67.82, 79.67, 112.30, 121.51, 121.66, 127.34, 127.88, 128.59, 129.93, 138.47, 154.71, 155.92.

### Binding Studies

#### Radioligand Binding Assays for 5-HT_1A_, 5-HT_6_, and 5-HT_7_ Receptors in Transfected HEK293 Cells

##### Cell Culture

HEK293 cells with stable expression of human 5-HT_1A_, 5-HT_6_, and 5-HT_7b_ (prepared with the use of Lipofectamine 2000) were grown in Dulbecco’s Modified Eagle Medium (DMEM) containing 10% dialyzed fetal bovine serum (FBS) and 500 μg/mL G418 sulfate. Liver hepatocellular carcinoma Hep-G2 cells were cultured in DMEM enriched by 10% FBS, 2 mM l-glutamine, 100 IU/mL penicillin, and 50 μg/mL streptomycin (all from Sigma-Aldrich Corporation, a division of Merck KGaA, Darmstadt, Germany). Cell lines were maintained at 37 °C in a humidified atmosphere with 5% CO_2_. For membrane preparation, the cells were subcultured in 150 cm^2^ flasks, grown to 90% confluence, washed twice with prewarmed to 37 °C phosphate buffered saline (PBS), and pelleted by centrifugation (200*g*) in PBS containing 0.1 mM EDTA and 1 mM dithiothreitol. Prior to membrane preparation, pellets were stored at −80 °C.

The cell pellets were thawed and homogenized in 10 volumes of assay buffer using an Ultra Turrax tissue homogenizer and centrifuged twice at 35 000*g* for 15 min at 4 °C, with incubation for 15 min at 37 °C in between. The composition of the assay buffers was as follows: for 5-HT_1A_R: 50 mM Tris HCl, 0.1 mM EDTA, 4 mM MgCl_2_, 10 μM pargyline, and 0.1% ascorbate; for 5-HT_6_R: 50 mM Tris HCl, 0.5 mM EDTA, and 4 mM MgCl_2_; for 5-HT_7b_R: 50 mM Tris HCl, 4 mM MgCl_2_, 10 μM pargyline, and 0.1% ascorbate. All the assays were incubated in a total volume of 200 μL in 96-well microtiter plates for 1 h at 37 °C, except 5-HT_1A_R which was incubated at room temperature. The process of equilibration was terminated by rapid filtration through Unifilter plates with a 96-well cell harvester and radioactivity retained on the filters was quantified on a Microbeta plate reader (PerkinElmer, USA). For the displacement studies, the assay samples contained the following radioligands (PerkinElmer, USA): 2.5 nM [^3^H]-8-OH-DPAT (135.2 Ci/mmol) for 5-HT_1A_R; 2 nM [^3^H]-LSD (83.6 Ci/mmol) for 5-HT_6_R; 0.8 nM [^3^H]-5-CT (39.2 Ci/mmol) for 5-HT_7_R. Nonspecific binding was defined with 10 μM of 5-HT in 5-HT_1A_R and 5-HT_7_R binding experiments, where 10 μM of methiothepine was used in 5-HT_6_R assays. Each compound was tested in triplicate at 7 concentrations (10^–10^–10^–4^ M). The results were analyzed with one-way ANOVA followed by the Bonferroni’s posthoc test. The inhibition constants (p*K*_i_) were calculated from the Cheng-Prusoff equation.^46^ The results were expressed as means of at least two separate experiments.

##### Radioligand Binding Assays for 5-HT_2A_ and 5-HT_2C_ Receptors on Rat Brain Membranes

Male Sprague–Dawley rats were decapitated and their brains removed and placed on ice. The hippocampi (for the functional 5-HT_1A_ receptor assay) or frontal cortexes (for the competitive 5-HT_2A_ and 5-HT_2C_ receptor assays) were dissected and homogenized with a glass homogenizer in 30 vol. ice-cold TED buffer (50 mMTris-HCl, 1 mM EDTA, 1 mM dithiotheritol, pH 7.4). Next, the homogenate was centrifuged at 21 000*g* for 30 min at 4 °C. The pellet was suspended in 30 vol TED buffer (pH 7.4) and incubated in a water bath for 10 min at 37 °C to remove endogenous ligands. The suspension was centrifuged again at 21 000*g* for 30 min at 4 °C. The pellet was resuspended in 30 vol TED buffer (pH 7.4) and the centrifugation step was repeated. The final pellet was suspended in 10 vol 50 mM Tris-HCl (pH 7.4) and stored at −80 °C until use.^47^

The 5-HT_2A_ assay was performed according to a previously described protocol with some modifications.^48^ Briefly, the frontal cortex homogenates (160 μg protein/mL) were incubated in triplicate with 1 nM [^3^H]ketanserin for 60 min at 36 °C in a 50 mM Tris-HCl (pH 7.4) buffer containing 0.1% ascorbate, 3 mM CaCl_2_, and 10 μM pargyline) and increasing concentrations (10^–11^–10^–5^ M) of the compound of interest. Nonspecific binding was determined in the presence of 10 μM mianserin. For the 5-HT_2C_ assay, the frontal cortex homogenates (250 μg protein/mL) were incubated in triplicate with 1 nM [^3^H]mesulergine for 60 min at 36 °C in a 50 mM Tris-HCl (pH 7.4) buffer containing 0.1% ascorbate, 10 mM MgCl_2_, 10 μM pargyline, 100 nM spiperone, and increasing concentrations (10^–10^–10^–5^ M) of the compound tested. After incubation, the reaction mixture was deposited onto UniFilter-96 GF/B plates, with the aid of a FilterMate-96 Harvester. The filter plates were presoaked with 0.4% PEI for 1 h. Next, each filter well was washed with 1.75 mL of 50 mM Tris-HCl (pH 7.4) and left to dry on a heating block set to 50 °C for 2 h. Then, 45 μL of Microscint-20 scintillation fluid was added to each filter well and left to equilibrate overnight. The filter-bound radioactivity was counted in a MicroBeta^2^ Microplate Counter. Curves were fitted with a one-site sigmoidal dose–response equation and p*K*_i_ for each compound was calculated with the Cheng-Pursoff equation. Results were analyzed with one-way ANOVA followed by the Bonferroni’s posthoc test. The results are expressed as means ± SEM of three independent experiments.

##### Functional 5-HT_1A_ Binding Assay

The [^35^S]GTPγS assay was performed according to the method described previously.^49^ In the agonist mode, 25 μg/mL of hippocampus homogenate was incubated in triplicate with 0.8 nM [^35^S]GTPγS in an assay buffer (50 mM Tris-HCl, pH = 7.4, 1 mM EGTA, 3 mM MgCl_2_, 100 mM NaCl, 30 μM GDP) in the presence of increasing concentrations of the tested compounds (10^–10^–10^–5^ M). Nonspecific binding was determined with 100 μM of unlabeled GTPγS. The reaction mixture was incubated for 90 min at 36 °C in a volume of 250 μL. To test whether G-protein stimulation was 5-HT_1A_ receptor dependent, in a separate experiment, the EC_80_ concentration of each compound was incubated with increasing concentrations (10^–10^–10^–5^ M) of WAY-100635. Next, 96-well Unifilter Plates (PerkinElmer, USA) were presoaked for 1 h with 50 mM Tris-HCl (pH = 7.4) before harvesting. The reaction was terminated by vacuum filtration onto filter plates with the Filter Mate Harvester (PerkinElmer, USA). The samples were then rapidly washed with 2 mL of 50 mM Tris-HCl (pH = 7.4) buffer. The filter plates were dried for 2 h at 50 °C. After drying, 45 μL of EcoScint-20 scintillant (PerkinElmer, USA) was added to every well. The radioactivity was counted in a Trilux MicroBeta^2^ counter (PerkinElmer, USA). The data were analyzed with GraphPad Prism 5.0 software (GraphPad Software, San Diego California USA, www.graphpad.com). To determine whether compound-stimulated [^35^S]GTPγS binding involved 5-HT_1A_ receptors, two protocols were used. In the first experiment, a single WAY-100635 concentration (10^–7^ M) was used against increasing concentrations of the agonist (10^–10^–10^–5^ M) to determine the rightward shift in EC_50_ (see Table SI-1). In the second protocol, increasing concentrations of WAY-100635 (10^–10^–10^–5^ M) were used against a single EC_80_ concentration of the compound studied to check if [^35^S]GTPγS binding was 5-HT_1A_-dependent (see Table SI-1).

The curves were fitted with a one-site nonlinear regression model. Efficacy (*E*_max_) and potency (pEC_50_) were calculated from the Cheng-Prusoff equation from three separate experiments and expressed as the means ± SEM. The drugs were classified as full or partial agonists based on their *E*_max_ values. Compounds that produced half of the stimulation for the endogenous full agonist serotonin were considered as partial agonists.^50^ The differences in compound potency and efficacy were evaluated with one-way ANOVA followed by the Bonferroni’s post-hoc test. The baseline G-protein stimulation was set at 100%. One, two, or three symbols represent statistical significance of 0.05, 0.01, and 0.001, respectively.

### Molecular Modeling

#### Ligand Preparation

All the herein investigated couples of enantiomers were built, parametrized (Gasteiger-Hückel method), and energy minimized, within MOE using an MMFF94 force field (the root-mean-square gradient has been set to 0.00001) [MOE: Chemical Computing Group Inc., Montreal, H3A 2R7 Canada. http://www.chemcomp.com].^51^

#### Homology Modeling and Docking Studies

As most of the key residues characteristic of GPCRs are conserved within the serotoninergic system, a novel 5-HT_1A_ receptor homology model was generated, taking into account the X-ray structure of human 5-HT_1B_ (PDB code: 5V54; resolution = 3.9 Å), in complex with methiotepin.^30^ This model was built by applying the ligand-based homology modeling strategy, as proposed by Moro, in order to take into account the role played by the ligand placed within the receptor crevice.^52^ This method was previously performed and discussed by us, for building other protein theoretical models.^53,54^ In this case, during the homology modeling calculations, we maintained the methiothepin structure, being the cocrystallized compound at the template cavity. Notably, methiothepin is a nonselective serotoninergic ligand, opening the possibility of building a more reliable model of the biological target, rather than taking into account only the coordinates of the template. The amino acid sequence of 5-HT_1A_R (P08908) was retrieved using the swissprot databank,^55^ while the three-dimensional structure coordinates file of the GPCR template was downloaded from the Protein Data Bank.^56^ The amino acid sequence of the biological target 5-HT_1A_R was aligned with the corresponding residues of 5V54, on the basis of the Blosum62 matrix (MOE software), obtaining a high value of the pairwise percentage residue identity (PPRI = 53%). The connecting loops were constructed by the loop search method implemented in MOE. The MOE output file included a series of ten models for the 5-HT_1A_ protein, independently built on the basis of a Boltzmann-weighted randomized procedure^57^ combined with specialized logic for the handling of sequence insertions and deletions.^58^ Among the derived models, there were no significant main chain deviations. The best scored model as packing quality functions was selected for a further full energy minimization, using the AMBER94 force field.^59^ Then, the putative binding site of 5-HT_1A_ targeting ligands was identified based on the alignment onto the known binding site of the serotoninergic template cocrystallized with methiothepin. This approach allowed us to identify the protein crevice involved in the ligand binding, as we previously successfully discussed for other GPCRs.^60−63^ Docking calculations of each enantiomer were then performed by means of LeadIT 2.1.8 software suite (www.biosolveit.com). The final docking poses were prioritized using the score values of the lowest energy pose of the compounds docked to the protein structure. All the ligands were refined and rescored by assessment with the algorithm HYDE, included in the LeadIT 2.1.8 software. The HYDE module considers dehydration enthalpy and hydrogen bonding.^64,65^

### Analysis of Cell Viability

Liver hepatocellular carcinoma Hep-G2 cells were seeded in 96-well plates (1 × 10^4^ cells/well) and maintained under continuous stimulation with increasing concentrations (nM−μM range) of the tested compounds. 200 mM ethanol and 5% Triton X-100 (Sigma-Aldrich Corporation) served as controls. The viability of cells was assessed by MTT assay as previously described.^66,67^ The absorbance was detected by a Victor3 plate reader (PerkinElmer Inc., Waltham, MA, USA) and represented by box and whiskers plot, as a measure of cell viability. Differences between values from treated and untreated cells were evaluated by two-ANOVA followed by Dunnett’s multiple comparison test (*p* < 0.05).

### CYL8038QP2 hERG Human Potassium Ion Channel Cell Based QPatch CiPA Assay

After whole cell configuration is achieved, the cell is held at −80 mV. A 500 ms pulse to −40 mV is delivered to measure the leaking current, which is subtracted from the tail current online. Then, the cell is depolarized to +40 mV for 500 ms and then to −80 mV over a 100 ms ramp to elicit the hERG tail current. This paradigm is delivered once every 8 s to monitor the current amplitude.

## Data Calculation and Analysis

The parameters measured were the maximum tail current evoked on stepping to 40 mV and ramping back to −80 mV from the test pulse. All data were filtered for seal quality, seal drop, and current amplitude. The peak current amplitude was calculated before and after compound addition, and the amount of block was assessed by dividing the Test compound current amplitude by the Control current amplitude. Control is the mean hERG current amplitude collected 15 s at the end of the control; Test Compound is the mean hERG current amplitude collected in the presence of test compound at each concentration.

E-4031, a blocker of hERG-type potassium channels, was used as reference compound.

### Behavioral Tests

#### Formalin Test

Male Swiss CB1 mice (Envigo, S. Pietro al Natisone (UD)) weighing 25–30 g were used. The animals were kept at a constant room temperature (25 ± 1 °C) under a 12:12 h light and dark cycle, with free access to food and water. Each mouse was used for only one experiment. The experimental procedures were conducted in accordance with international guidelines, as well as the European Communities Council Directive and National Regulations (CEE Council 86/609 and DL 116/92). All the tests were performed blind to treatment.

The formalin (5%, 10 μL; Sigma-Aldrich) was injected subcutaneously into the plantar side of the right hind paw.^68^ After injection, the mice were immediately placed in a plexiglas box: the total time (in seconds) spent on licking or biting the injected hind paw was recorded every 5 min at selected intervals, 0–10 (phase I) and 10–60 (phase II) min, in the different experimental groups, as an indicator of nociceptive behavior. The formalin scores were separated into two phases: phase I (0–10 min) and phase II (10–60 min). A mean response was then calculated for each phase. The test compound and WAY-100635 (Sigma-Aldrich) were dissolved in normal saline solution, containing 10% dimethyl sulfoxide (DMSO, Sigma-Aldrich). A vehicle solution containing 10% DMSO was given as a control. Morphine was used as positive control. The test compound and vehicle were intraplantar (i.p.) administered (5 mL/kg) 15 min before the formalin. The WAY-100635 (3 mg/kg i.p.) was injected 30 min before the test compound or vehicle. The data are expressed as mean values (SEM). Analyses of variance (two-way repeated measures ANOVA followed by post hoc Bonferroni test) were performed to assess significance using the Instat 3.0 software (GraphPad Software, San Diego, CA). *p* < 0.05 was considered significant.

#### Hot Plate Test

The experiments were performed on male Albino Swiss mice (20–25 g), where 4 animals were kept in a cage, in an environmentally controlled room (ambient temperature 22 ± 1 °C; relative humidity 50–60%; 12 h light/dark cycle, lights on at 8:00). Standard laboratory food (LSM, Agropol-Motycz, Poland) and filtered water were available *ad libitum*. All the behavioral experiments were carried out in accordance with the European Community Council Directive for Care and Use of Laboratory Animals (2010/63/EU) (2016), and approved by the Local Ethics Committee for Animal Experimentation. The tested compounds were administered intraperitoneally (i.p.), dissolved in DMSO (its final concentration of 0.1%), suspended in 0.5% Tween-80 (1–2 drops) and then diluted using an aqueous solution of 0.5% methylcellulose (tylose). All the compounds were given in a manner generally accepted in experimental pharmacology, in a volume of 10 mL/kg body weight. The animals were weighed immediately before injection. Each group consisted of 8 members. The control animals received an equivalent volume of the solvent at the respective time before the tests. Morphine was used as positive control. Between injections, the mice were provided with stable living conditions and unrestricted access to food and water. All the experiments were conducted in the light phase between 09.00 a.m. and 05.00 p.m.

For the hot plate test, the mice were placed on a hot plate (Ugo Basile Srl, Gemonio, Italy) maintained at a constant temperature of 55 °C with a cutoff time of 20 s. The baseline latency response (in seconds), induced by the thermal stimulus, with the mouse lifting either of the hind paws or jumping with all four feet off the hot plate, before administration of a compound was measured first. The animals were then administered with compounds and the post-treatment latency responses were determined at 30 min time intervals up to 180 min after drug injection. The antinociceptive effects of the compounds were expressed as a percentage of maximum possible effect (%MPE), which was calculated according to the following equation: [(*T*_1_ – *T*_0_)/(20 – *T*_0_)] × 100, where *T*_0_ and *T*_1_ are the predrug and post-drug latencies for hot plate response, respectively.^37,38^ The results were calculated using the one-way analysis of variance (ANOVA) followed by Bonferroni’s *post hoc* test. The results are presented as the means ± standard errors of mean (S.E.M.). The level of *p* < 0.05 was considered statistically significant. All the figures were prepared using the GraphPad Prism ver. 5.00 for Windows (GraphPad Software, San Diego, CA, USA), www.graphpad.com.

#### Preparation of Spinal Cord Slices and Patch-Clamp Recording

The Italian Ministry of Health approved all the experiments that were conducted on postnatal CD1 mice (P18–P25) in accordance with the Guide for the Care and Use of Laboratory Animals and the EU and Italian regulations on animal welfare. The spinal cord slices were obtained following the procedure described previously.^69,70^ Briefly, the animals were anesthetized with isoflurane and decapitated, the spinal cord and vertebrae were rapidly removed and placed in ice-cold dissecting Krebs’ solution (composition in mM:125 NaCl, 2.5 KCl, 1.25 NaH_2_PO_4_, 26 NaHCO_3_, 25 glucose, 6 MgCl_2_, 1.5 CaCl_2_, and 1 kynurenic acid, pH 7.4, 320 mOsm), bubbled with 95% O_2_, 5% CO_2_. The lumbar spinal cord was isolated, embedded in an agarose block (low melting point agarose 3%, Thermo Fisher Scientific, Waltham, USA), and transverse slices (500 μm thick) were obtained using a vibrating microtome (WPI, Sarasota, USA). The slices were incubated in oxygenated incubation Krebs’ solution (the same as dissecting but without kynurenic acid) at 35 °C for 30 min and used for recording. The Patch-clamp recording in whole-cell configuration was performed on lamina I–II neurons at room temperature. The slices were perfused at 2 mL/min with recording Krebs’solution (in mM: 125 NaCl, 2.5 KCl, 1.25 NaH_2_PO_4_, 26 NaHCO_3_, 25 glucose, 1 MgCl_2_, and 2 CaCl_2_, pH 7.4, 320 mOsm). Recordings of post-synaptic currents (EPSCs) were performed in voltage clamp, by using an intracellular solution with the following composition (in mM): 120 potassium methanesulfonate, 10 NaCl, 10 EGTA, 1 CaCl_2_, 10 HEPES, 5 ATP-Mg, pH adjusted to 7.2 with KOH, osmolarity 300 mOsm. 8-OH-DPAT was obtained from Sigma-Aldrich (Saint Louis, USA). The data were recorded and acquired using a Multiclamp 700A amplifier and the pClamp 10 software (Molecular Devices, Sunnyvale, USA). The sampling rate was 10 kHz, and the data were filtered at 2 kHz. The data were analyzed off-line using pClamp10 software and Minianalysis (Synaptosoft, USA). The data are expressed as the mean ± SEM and differences were considered significant for *p* < 0.05. The statistical significance for the effect on EPSC frequency was determined using the Kolmogorov–Smirnov test on individual neurons.

## Supporting Information Available

Experimental Section: (i) Effect of a single/increasing concentrations of WAY-100635 on compound-stimulated [^35^S]GTPγS binding (Table SI-1/SI-2); (ii) Molecular modeling: Alignment of the 5HT_1A_ primary sequence with that of the template 5HT_1B_ (Figure SI-1); Superimposition of the modeled 5-HT_1A_ receptor onto the X-ray crystallographic structure of the 5-HT_1B_ template (Figure SI-2); Match of the conserved regions among the modeled 5-HT_1A_ receptor and the X-ray crystallographic structure of the 5-HT_1B_ template (Figure SI-3); Positioning of the antagonist methiothepin at the modeled 5-HT_1A_R (Figure SI-4) (PDF)

## Abbreviations Used

5-HT: serotonin
SAR: structure–activity relationship
GTPγS: guanosine 5′-*O*-[gamma-thio]triphosphate
TMS: tetramethylsilane
