# Supplementary material for: Identification of a Potent and Selective 5-HT1A Receptor Agonist with In Vitro and In Vivo Antinociceptive Activity
Source: ACS Chem Neurosci. 2020 Dec 2;11(24):4111–27. doi: 10.1021/acschemneuro.0c00289 (PMC8016166; doi:10.1021/acschemneuro.0c00289)
Supplement: Supplementary file 1 — cn0c00289_si_001.pdf [file cn0c00289_si_001.pdf]

Identification of a potent and selective 5-HT1A receptor agonist with *in vitro* and *in vivo* antinociceptive activity

Pasquale Linciano,[1] Claudia Sorbi,[1] Antonella Comitato,[2] Anna Lesniak,[3] Magdalena Bujalska-Zadrożny,[3] Agata Pawłowska,[3] Anna Bielenica,[4] Jolanta Orzelska-Górka,[5] Ewa Kędzierska,[5] Grażyna Biała,[5] Simone Ronsisvalle,[6] Silvia Limoncella,[7] Livio Casarini,[7,8] Elena Cichero,[9] Paola Fossa,[9] Grzegorz Satała,[10] Andrzej J. Bojarski,[10] Livio Brasili,[1] Rita Bardoni,[2]* Silvia Franchini.[1]*

[1]*Department of Life Sciences, University of Modena and Reggio Emilia, Via Campi 103, 41125 Modena, Italy.*
[2]*Department of Biomedical, Metabolic and Neural Sciences, University of Modena and Reggio Emilia, Via Campi 287, 41125 Modena, Italy*
[3]*Department of Pharmacodynamics, Faculty of Pharmacy, Centre for Preclinical Research and Technology, Medical University of Warsaw, Banacha 1, 02-097 Warsaw, Poland*
[4] *Department of Biochemistry, Medical University of Warsaw, Banacha 1, 02-097 Warsaw, Poland*
[5]*Department of Pharmacology and Pharmacodynamics, Faculty of Pharmacy with Division of Medical Analytics, Medical University of Lublin, Chodzki 4A, 20-093 Lublin, Poland*
[6]*Department of Drug Sciences, Medicinal Chemistry Section, University of Catania, Viale A. Doria 6, I-95125 Catania, Italy.*
[7]*Unit of Endocrinology, Dept. Biomedical, Metabolic and Neural Sciences, University of Modena and Reggio Emilia, via G. Campi 287, 41125 Modena, Italy.*
[8]*Center for Genomic Research, University of Modena and Reggio Emilia, via G. Campi 287, 41125 Modena, Italy.*
[9]*Department of Pharmacy, Medicinal Chemistry Section, School of Medical and Pharmaceutical Sciences, University of Genova, Viale Benedetto XV 3, 16132 Genova, Italy.*
[10]*Department of Medicinal Chemistry, Maj Institute of Pharmacology, Polish Academy of Sciences, 12, Smętna Street, 31-343, Kraków, Poland*

Table of Content

Table SI-1. Effect of a single concentration of WAY100635 on compound-stimulated [35S]GTPγS binding in rat frontal cortex homogenates SI-2

Table SI-2. Effect of increasing concentrations of WAY100635 on compound-stimulated [35S]GTPγS binding in rat frontal cortex homogenates SI-3

Figure SI-1. Alignment of the $5HT_{1A}$ primary sequence with that of the template $5HT_{1B}$ …SI-4

Figure SI-2. Superimposition of the modelled 5-HT_{1A} receptor onto the X-Ray crystallographic structure of the 5-HT_{1B} template …SI-5

Figure SI-3. Match of the conserved regions among the modelled 5-HT_{1A} receptor and the X-Ray crystallographic structure of the 5-HT_{1B} template …SI-6

Figure SI-4. Positioning of the antagonist methiothepin at the modelled 5-HT_{1A}R …SI-7

Table SI-1. Effect of a single concentration of WAY100635 on compound-stimulated [35S]GTPγS binding in rat frontal cortex homogenates.

**Compound**	**pEC_{50} ± SEM**	**shift ratio**	**E_{max} (%) ± SEM**
rac-1	7.0 ± 0.2		151 ± 4.2
WAY + *rac*-1	6.2 ± 0.05 ***	6.3	162 ± 1.4
(*S*)-1	7.8 ± 0.22		138 ± 2.9
WAY + (*S*)-1	6.2 ± 0.11 ***	39.8	139 ± 2.2
(*R*)-1	7.2 ± 0.18		158 ± 5.9
WAY + (*R*)-1	6.2 ± 0.12 ***	10.0	168 ± 4.5
rac-2	7.4 ± 0.09		184 ± 2.7
WAY + *rac*-2	5.9 ± 0.09 ***	31.6	179 ± 3.4
(*S*)-2	7.6 ± 0.11		175 ± 2.7
WAY + (*S*)-2	6.2 ± 0.11***	25.1	171 ± 3.1
(*R*)-2	7.2 ± 0.21		143 ± 3.6
WAY + (*R*)-2	5.9 ± 0.07 ***	19.9	152 ± 3.0
8-OH-DPAT	7.5 ± 0.11		152 ± 2.4
WAY + 8-OH-DPAT	6.2 ± 0.16 ***	19.9	162 ± 2.4

Frontal cortex homogenates were incubated with the compound alone and compound + WAY - WAY100635 (10^{-7} M, WAY).) Results were expressed as means ± SEM. Curves were fitted with a one-site sigmoidal dose-response equation and potency (EC_{50}) and efficacy (E_{max}) were determined from 2 independent experiments. The pEC_{50} ratio was calculated as the ratio of pEC_{50} in the presence of WAY100635 divided by the pEC_{50} of agonist alone. Basal 5-HT_{1A}R activation was set to 100%. Results were analyzed with one-way ANOVA followed by the Bonferroni's post-hoc test. Comparisons were depicted as follows: *compound vs. compound + WAY100635. *** $p < 0.001$. [M] – molar concentration.

Table SI-2. Effect of increasing concentrations of WAY100635 on compound-stimulated [^{35}S]GTPγS binding in rat frontal cortex homogenates.

Compound	E_{max} (%) ± SEM
rac -1	138 ± 28.4
WAY + *rac* -1	90 ± 11.2 **
(*S*)-1	134 ± 24.5
WAY + (*S*)-1	103 ± 9.2 **
(*R*)-1	149 ± 35.1
WAY + (*R*)-1	90 ± 8.7 ***
rac-2	158 ± 24.6
WAY + *rac*-2	104 ± 13.5***
(*S*)-2	156 ± 21.7
WAY + (*S*)-2	104 ± 11.3***
(*R*)-2	140 ± 18.8
WAY + (*R*)-2	99.9 ± 9.6***
**8-OH-DPAT**	142 ± 19.4
**WAY + 8-OH-DPAT**	98 ± 7.5***

Frontal cortex homogenates were incubated with the EC_{80} compound concentration and increasing concentrations of WAY100635 (10^{-10} M - 10^{-5} M, WAY). Results were expressed as means ± SEM. Curves were fitted with a one-site sigmoidal dose-response equation and Emax (%) of the EC_{80} agonist concentration alone and in the presence of WAY100635 were determined from 2 independent experiments. Basal (no ligand added) [^{35}S]GTPγS binding was set to 100%. Results were analyzed with one-way ANOVA followed by the Bonferroni's post-hoc test. None of the values were significantly different from basal [^{35}S]GTPγS binding. *compound vs. compound + WAY100635. ** $p < 0.01$; *** $p < 0.001$.

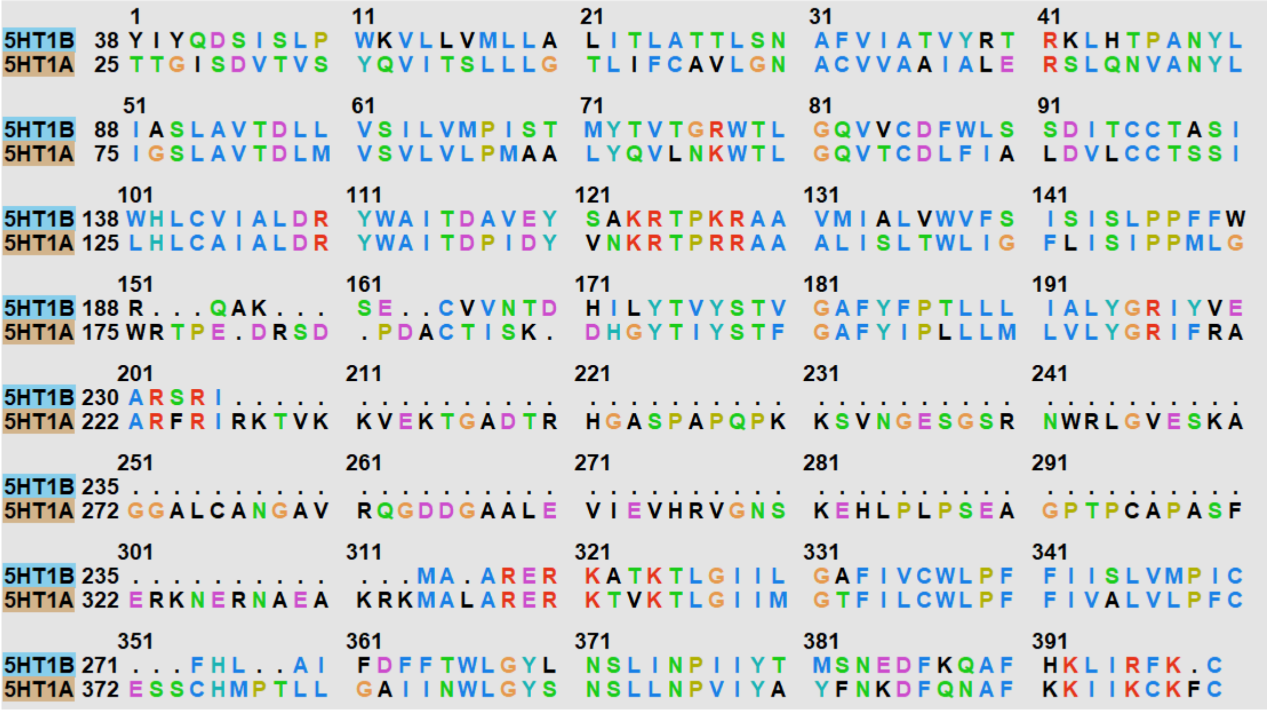

Figure SI-1: Alignment of the $5HT_{1A}$ primary sequence with that of the template $5HT_{1B}$. Positively- and negatively-charged residues are labelled in red and pink, respectively. Hydrophobic and polar aminoacids are shown in blue and green.

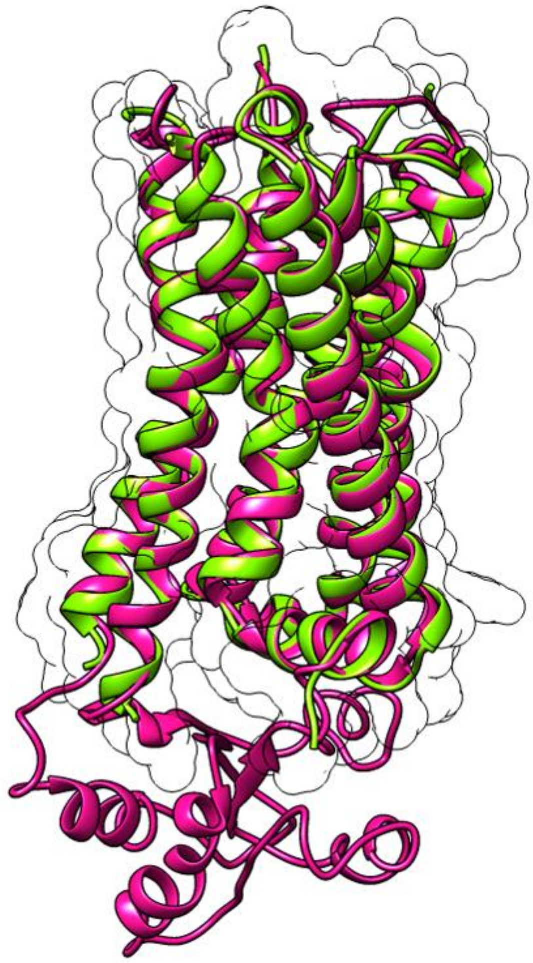

Figure SI-2. Superimposition of the modelled 5HT-$_{1A}$ receptor (magenta) onto the X-Ray crystallographic structure of the 5HT-$_{1B}$ template (green).

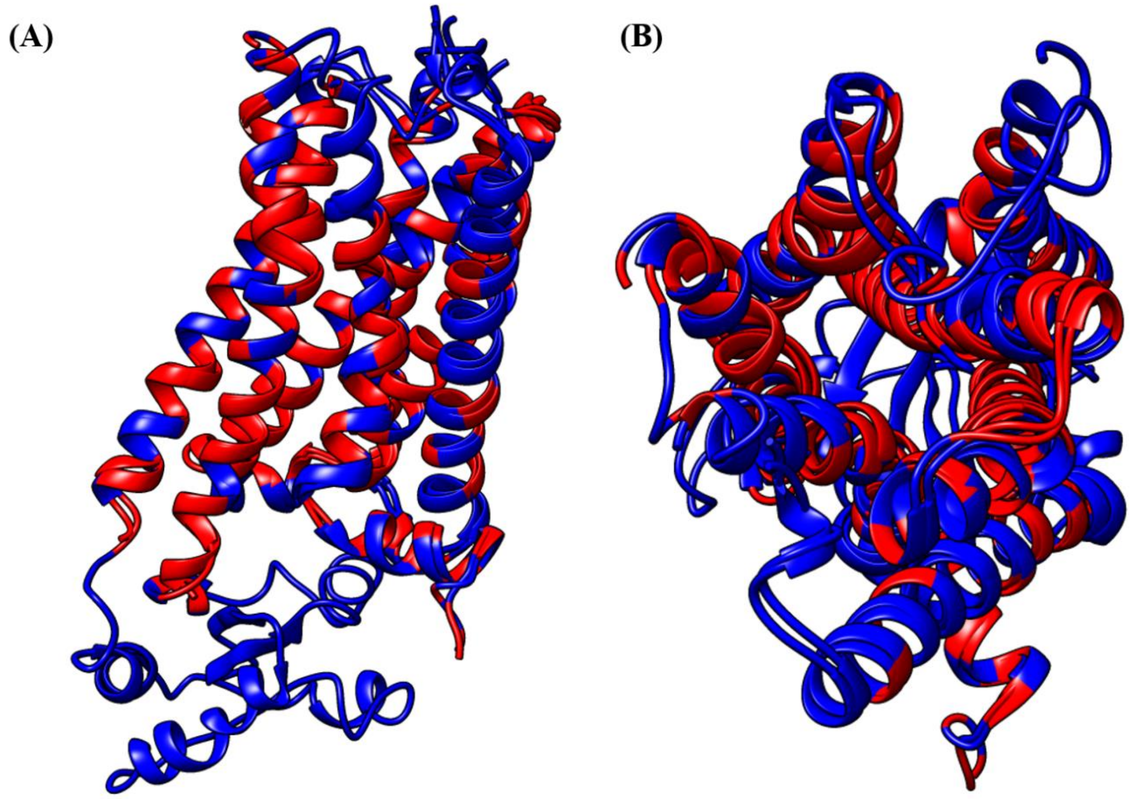

Figure SI-3. Match of the conserved regions among the modelled 5-HT_{1A} receptor and the X-Ray crystallographic structure of the 5-HT_{1B} template shown as side-view (**A**) and top view (**B**). Conserved regions are highlighted as red ribbon.

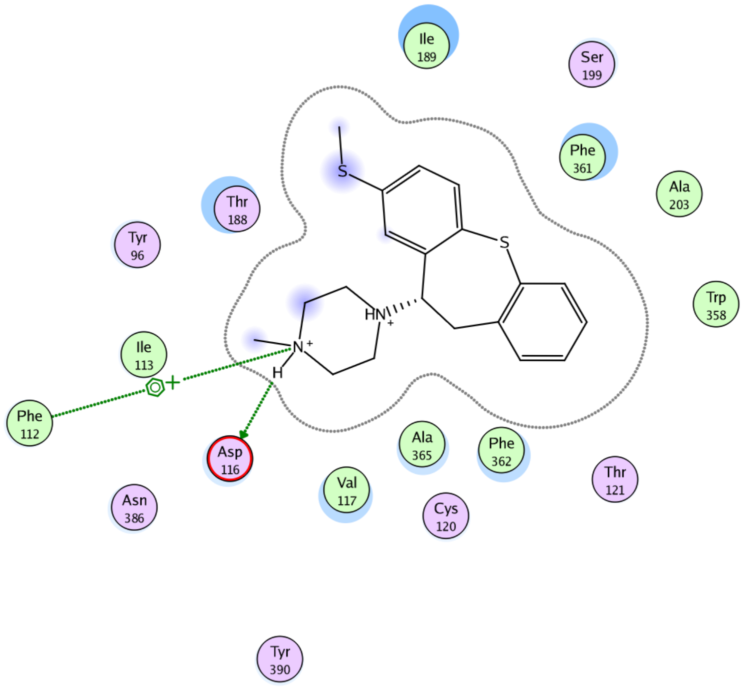

Figure SI-4. Positioning of the antagonist methiothepin at the modelled 5-HT_{1A}R. Polar and hydrophobic residues are colored in pink and green, respectively.
